# Gender Differences in Global but Not Targeted Demethylation in iPSC Reprogramming

**DOI:** 10.1016/j.celrep.2017.01.008

**Published:** 2017-01-31

**Authors:** Inês Milagre, Thomas M. Stubbs, Michelle R. King, Julia Spindel, Fátima Santos, Felix Krueger, Martin Bachman, Anne Segonds-Pichon, Shankar Balasubramanian, Simon R. Andrews, Wendy Dean, Wolf Reik

**Affiliations:** 1 Epigenetics Programme, The Babraham Institute, Cambridge CB22 3AT, UK; 2 Bioinformatics Group, The Babraham Institute, Cambridge CB22 3AT, UK; 3 Department of Chemistry, University of Cambridge, Cambridge CB2 1EW, UK; 4 Cancer Research UK Cambridge Institute, University of Cambridge, Cambridge CB2 0RE, UK; 5 Centre for Trophoblast Research, University of Cambridge, Cambridge CB2 3EG, UK; 6 Wellcome Trust Sanger Institute, Hinxton CB10 1SA, UK

**Keywords:** reprogramming, iPSCs, DNA methylation, gender differences, AID, UHRF1

## Abstract

Global DNA demethylation is an integral part of reprogramming processes in vivo and in vitro, but whether it occurs in the derivation of induced pluripotent stem cells (iPSCs) is not known. Here, we show that iPSC reprogramming involves both global and targeted demethylation, which are separable mechanistically and by their biological outcomes. Cells at intermediate-late stages of reprogramming undergo transient genome-wide demethylation, which is more pronounced in female cells. Global demethylation requires activation-induced cytidine deaminase (AID)-mediated downregulation of UHRF1 protein, and abolishing demethylation leaves thousands of hypermethylated regions in the iPSC genome. Independently of AID and global demethylation, regulatory regions, particularly ESC enhancers and super-enhancers, are specifically targeted for hypomethylation in association with transcription of the pluripotency network. Our results show that global and targeted DNA demethylation are conserved and distinct reprogramming processes, presumably because of their respective roles in epigenetic memory erasure and in the establishment of cell identity.

## Highlights

• iPSC reprogramming induces transient genome-wide DNA demethylation
• Global demethylation is more pronounced in female iPSCs
• AID is a critical regulator of UHRF1 protein and genome-wide DNA demethylation
• Targeted DNA demethylation occurs at key regulatory regions and is uncoupled from global demethylation

## Graphical Abstract

**Figure:**
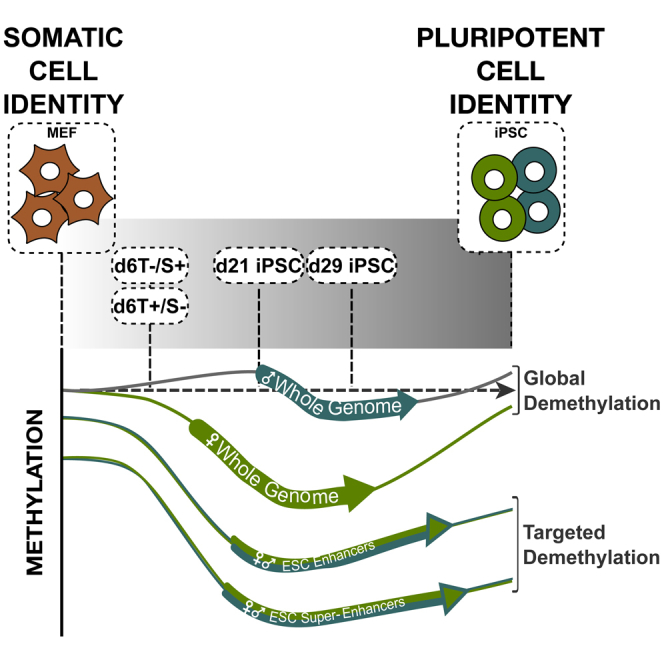

## Teaser

Milagre et al. find that two modes of DNA demethylation occur during primary iPSC reprogramming. Global DNA demethylation, more pronounced in female cells, is regulated by AID through UHRF1 and occurs transiently at intermediate-late stages of reprogramming. Targeted DNA demethylation, by contrast, is important in establishing hypomethylation at enhancers of pluripotency genes and occurs similarly in female and male cells.

## Introduction

Induced pluripotent stem cell (iPSC) technology holds unparalleled promise for research, tissue engineering, and regenerative medicine. Reprogramming is a continuous process characterized by the stepwise activation of fundamental pluripotency genes (Brambrink et al., 2008, Stadtfeld et al., 2008) and the silencing of somatic cell-of-origin genes (Buganim et al., 2012). This complex remodeling of transcriptional networks is associated with reprogramming of the epigenome (Brambrink et al., 2008, Sridharan et al., 2009, Stadtfeld et al., 2008), which is the ensemble of DNA or chromatin modifications linked with gene expression states without affecting DNA sequence. Specific histone marks are lost (Chen et al., 2013b, Golipour et al., 2012, Yang et al., 2014) or acquired (Buganim et al., 2012, Cacchiarelli et al., 2015, Mikkelsen et al., 2008) during reprogramming, and activation of microRNAs (Polo et al., 2012) and long noncoding RNAs (Kim et al., 2015) at defined stages is also important. A critical role for DNA demethylation in complete and robust reprogramming of terminally differentiated cells has been proposed (Papp and Plath, 2013); however, the molecular mechanisms underlying this epigenetic process and its dynamics at different stages during reprogramming are poorly understood. It is also not clear to what extent demethylation is involved in the creation of a pluripotent cell identity and whether it may also be needed to remove epigenetic memory.

iPSCs have been shown to tolerate global hypomethylation, just as embryonic stem cells (ESCs) do (Wernig et al., 2007), and the efficiency of obtaining these cells is improved by treatment with 5-azacytidine (Mikkelsen et al., 2008). Tet family dioxygenases hydroxylate 5-methylcytosine (5mC) and enhance reprogramming efficiency (Costa et al., 2013, Doege et al., 2012, Hu et al., 2014). The cytosine deaminase activation-induced cytidine deaminase (AID) stabilizes the pluripotent phenotype (Kumar et al., 2013) and is needed for demethylation of specific promoters in heterokaryon reprogramming (Bhutani et al., 2010), but the extent, timing, and mechanisms of this demethylation in iPSC reprogramming are not known.

Despite demethylation being critical, there are only a few comprehensive studies of DNA methylation dynamics during the reprogramming process and none in a primary reprogramming system. Polo et al. (2012), using a methylation array to study promoter regions, showed that demethylation occurs gradually, while new methylation marks are gained only late in reprogramming. More recently, Lee et al. (2014a) performed genome-wide analyses in intermediates of F-class cells (an alternative pluripotent state, dependent on continuous high expression levels of the Yamanaka Factors [Oct3/4, Sox2, Klf4, cMyc] OSKM) and one late time point where OSKM were no longer exogenously expressed. They demonstrated that hypomethylated differentially methylated regions (DMRs) are highly enriched in H3K4me3 and H3K27me3 and that the majority of these overlap with specific transcription factor binding sites. However, it is not clear how DNA methylation marks at regulatory regions are remodeled during primary iPSC reprogramming and how this is linked to the establishment of the pluripotent gene expression program. Here, we perform a comprehensive genome-wide in-depth analysis of the dynamics of DNA demethylation and its link to transcription during primary mouse embryonic fibroblast (MEF) reprogramming to iPSCs. We demonstrate that both female and male cells undergo global hypomethylation of the genome, which is likely to be important for the removal of epigenetic memory. Independently, targeted loss of DNA methylation marks at critical regulatory regions is necessary for the establishment of cell identity. We show that global demethylation is more pronounced in female cells, while targeted demethylation at regulatory regions is evident in both female and male cells. Finally, we find that AID plays a key role in global demethylation and epigenetic memory erasure. Surprisingly, this occurs at the level of regulation of UHRF1 protein, an essential component of the DNA methylation maintenance machinery, recently also shown to be regulated during global demethylation in ESCs transitioning from serum to 2i (von Meyenn et al., 2016).

## Results

### iPSC Reprogramming Triggers Transient Global DNA Hypomethylation

**Figure 1:**
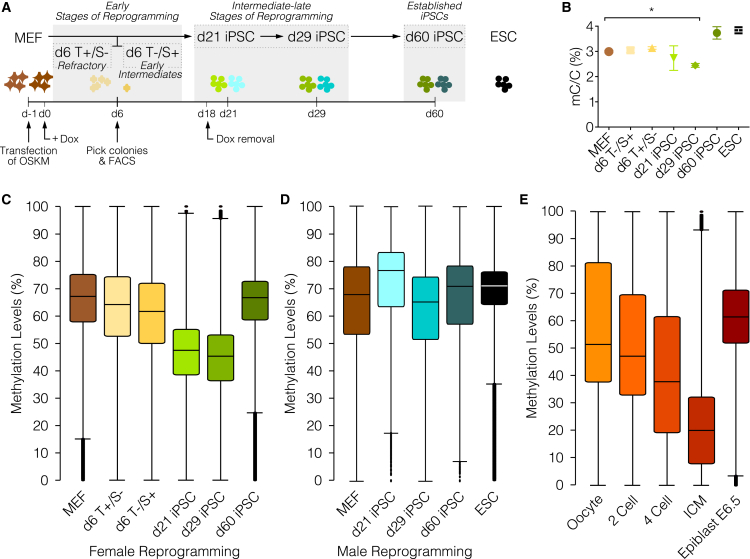
Global DNA Demethylation Dynamics during iPSC Reprogramming (A) Scheme of the reprogramming system. d6T^+^/S^–^ and d6T^–^/S^+^: Thy1 (T) and SSEA1 (S) FACS cells at day 6 of reprogramming. For detailed description, refer to Experimental Procedures. (B) Global 5mC levels measured by LC-MS. Results are expressed as percentage of total cytosines and data are represented as mean ± SEM p values shown are the result of an ANOVA with Dunnett’s correction. (C–E) CpG methylation levels, as assessed by BS-seq, during reprogramming of (C) female, (D) male cells, and (E) pre-implantation embryo (oocyte, 2- and 4-cell embryos, ICM, and epiblast). (C–E) Plot displays the median (bar), inter-quartile range (box), and maximum and minimum (whiskers). See also Figure S1.

To enable dynamic methylation profiling, reprogramming intermediates were analyzed at defined time points (Figure 1A; for a detailed description, see Experimental Procedures). Oct4-GFP MEFs were reprogrammed in low oxygen (5% O_2_), known to improve reprogramming (Yoshida et al., 2009), in serum medium and using an inducible piggybac system. This allowed us to obtain and pick colonies as early as day 6 (d6) after induction of OSKM by doxycycline (Dox) and analyzing these same clones over time, allowing for the characterization of intermediate time points that are not accessible through primary reprogramming carried out in normoxic conditions (Figure 1A). Female and male iPSC clones at intermediate-late stages of reprogramming (d21 and d29 iPSCs) already express the majority of the pluripotency factors, but in contrast to established iPSCs (d60 iPSCs) female cells are still in the process of downregulating Xist and thus in the process of completing X chromosome reactivation (Figure S1A). Dox-independent GFP-positive colonies at d21 that showed expression of key pluripotency markers and the ability to differentiate into the three germ layers as well as a normal karyotype by d60 (Figures S1B–S1D) were used in subsequent analyses. Embryonic stem cells (ESCs) were included in the analysis as a control for pluripotent cells.

Notably, liquid chromatography mass spectrometry (LC-MS) revealed substantial global demethylation in intermediate-late stages of reprogramming in female cells (Figure 1B). While methylation levels comparable to MEFs (3.0%) were maintained at d6 (d6T^+^/S^–^: 3.0% and d6T^–^/S^+^: 3.1%), in intermediate-late stages there was a significant decrease in 5-methylcytosine (5mC) levels (d21 iPSCs: 2.7% and d29 iPSC: 2.4%, p < 0.05: MEFs versus d29 iPSCs, ANOVA with Dunnett’s correction). However, this global hypomethylation was transient with established iPSCs’s 5mC levels (3.8%) similar to those of primed ESCs (3.9%). Global demethylation during reprogramming of female cells was confirmed by whole-genome bisulphite sequencing (WGBS), which revealed a substantial drop in CpG methylation levels from 68% in MEFs to 45% in d29 iPSCs, with subsequent remethylation to 67% in d60 iPSCs (Figures 1C, S1E, and S1G). Interestingly, during reprogramming of male cells this global demethylation was not as marked as in female cells (Figures 1D, S1F, and S1G).

The methylation dynamics during iPSC reprogramming closely resembled the transient loss of DNA methylation marks in early embryo development, where DNA methylation is globally lost from the oocyte (52%) to the 2- and 4-cell stage (47% and 38%) until it reaches very low levels (20%) in the inner cell mass (ICM) at the blastocyst stage. Methylation marks are then regained by the E6.5 epiblast stage (61%) (Figures 1E and S1H). Moreover, global demethylation was uncoupled from transcriptional regulation (Figure S1I) as previously reported in primordial germ cell (PGCs) development, and in the transition from primed to naive ESCs (Seisenberger et al., 2012, Ficz et al., 2013).

These results show that global DNA demethylation occurs in the intermediate-late stages of reprogramming and that female and male cells undergo different modulation of DNA methylation dynamics during reprogramming.

### Stable Targeted DNA Demethylation Occurs at Pluripotency Regulatory Regions and Correlates with Expression of the Pluripotency Network

**Figure 2:**
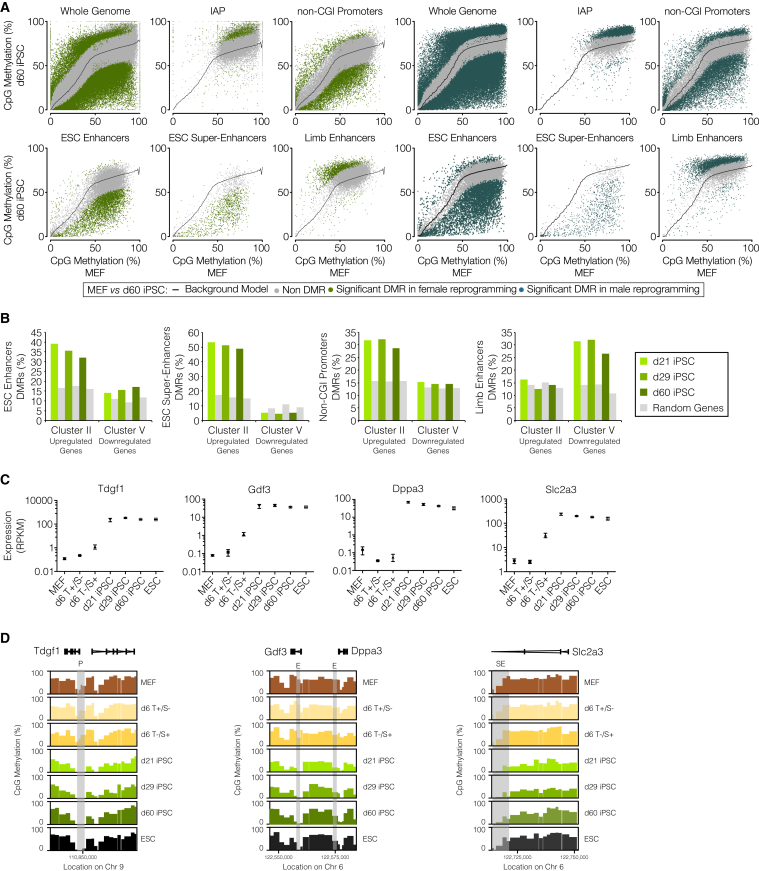
Correlation of DNA Methylation at Different Regulatory Regions and Gene Expression (A) Scatterplot of DNA methylation levels of individual probes genome-wide, showing whole genome and different genomic features of MEFs and d60 iPSCs. Dots represent individual 50 CpG probes—significant differentially methylated regions (DMRs) are represented in green (female cell reprogramming) or blue (male cell reprogramming). Background model depicted as a black line. (B) Percentage of DMRs at regulatory regions that overlap with specific gene clusters, compared to random sets with the same number of genes (in gray). (C) Expression profiles (reads per kilobase pre million mapped reads [RPKM]) for *Tdgf1*, *Gdf3*, *Dppa3*, and *Slc2a3*. (D) Example of BS-seq profile for *Tdgf1*, *Gdf3*, *Dppa3*, and *Slc2a3* at promoter, enhancer, and SE regions. Methylation levels of individual probes, between 0% and 100% are shown. Shadowed areas highlight promoter (P), enhancer (E), or super-enhancer (SE) regions. See also Figure S2.

In order to integrate and validate the differences in global methylation levels observed between the discrete time points within our experiment, the development of specific analytical approaches was required. These approaches also allowed insights into other reprogramming systems, thus highlighting their usefulness (for detailed description, see Supplemental Experimental Procedures). The first approach employs a background model to correct for global methylation differences, which allowed us to confidently call differentially methylated regions (DMRs) from MEFs to established iPSCs. Gene bodies, intergenic regions, and long interspersed nuclear elements (LINEs) and short interspersed nuclear elements (SINEs) have a similar methylation profile to the genome as a whole and hence follow the global demethylation and remethylation event (Figures 2A and S2A). In contrast, intracisternal A particle (IAP) retrotransposons and limb enhancers (as an example of a tissue-specific enhancer) are protected from demethylation, with many DMRs being hypermethylated in the established iPSCs. Notably, pluripotency regulatory regions such as promoters, ESC enhancers, and super-enhancers (SEs) are specifically targeted for demethylation, with the majority of these DMRs being hypomethylated over and above the genome average (Figures 2A and S2A). The importance of hypomethylation for enhancer and SE function in ESCs is well documented (Ding et al., 2015, Stadler et al., 2011, Wiench et al., 2011) but has not been described in iPSC reprogramming. To validate the DMRs found from WGBS, an amplicon-based assay was designed. This assay allowed the methylation dynamics of selected regions to be interrogated at >1,000-fold sequencing depth. The results closely matched those obtained from our low sequencing depth (3-fold) WGBS data, showing that such coverage nevertheless provided robust methylome information on individual loci (Figure S2B). Unlike global demethylation, targeted demethylation occurs at the same regions and to a similar extent during female and male somatic cell reprogramming (Figure 2A).

**Figure 3:**
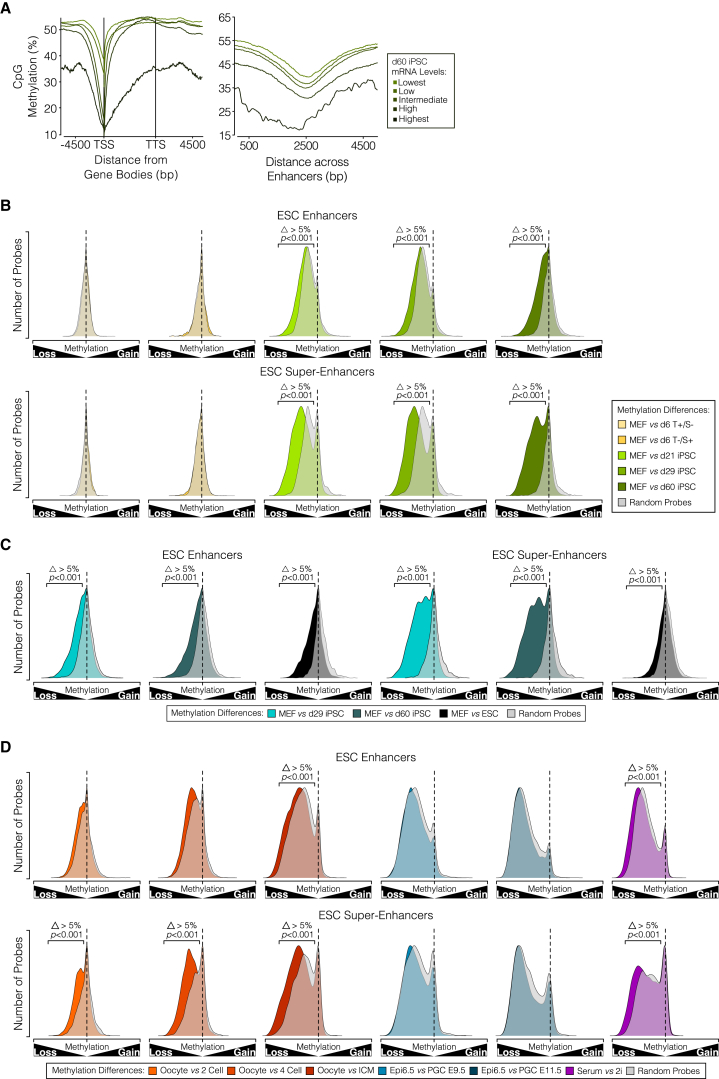
DNA Demethylation Dynamics at Specific Genomic Features and in Different Reprogramming Processes (A) CpG methylation levels for promoter and enhancer regions of genes showing different expression levels in female d60 iPSCs. (B and C) Density plots of methylation differences from MEFs to each time point in reprogramming of (B) female and (C) male cells and ESCs, at specific features shown by opaque plots, overlaid by MMRP transparent gray density plot. (D) Density plots of methylation differences at specific features, from oocyte to each time point in pre-implantation embryo, epiblast to PGCs and serum to 2i ESCs, shown by opaque plots; overlaid by MMRP transparent gray density plot. (B–D) Analyses were performed for ESC enhancers and SE Δ denotes a minimum 5% difference between data and MMRP profile. p values shown are the result of a pairwise t test with a Benjamini-Hochberg correction. See also Figure S3.

Hierarchical clustering of RNA sequencing (RNA-seq) data of female cells during reprogramming showed that differentially expressed genes from MEFs to established iPSCs fell into five distinct expression clusters (Figure S2C) similar to those previously described (O’Malley et al., 2013). Notably, genes in cluster II (upregulated during reprogramming—including the pluripotency network genes) are enriched for hypomethylated DMRs in non-CGI promoters, ESC enhancers, and SEs (Figure 2B). Conversely, limb enhancer DMRs, which remain hypermethylated, are absent from this cluster. In contrast, genes in cluster V (downregulated during reprogramming) are exclusively enriched for DMRs at limb enhancers (Figure 2B). Similar results were seen for d6T^–^/S^+^ and ESCs (Figure S2D), showing that upregulation of pluripotency genes precedes global demethylation and is influenced by targeted demethylation at ESC enhancers and super-enhancers. Instructive examples of changes in regulatory regions of individual genes are shown in Figures 2C, 2D, S2E, and S2F. These results show that demethylation at specific regulatory regions is important for the upregulation of the pluripotency network genes. In established female and male iPSCs, depletion of methylation in ESC enhancers and non-CGI promoters was more pronounced in highly and very highly expressed genes (Figure 3A), in agreement with previous studies in ESCs (Lister et al., 2009, Stadler et al., 2011).

We developed a second analytical approach that utilizes methylation-matched random probes (MMRPs) to account for global methylation differences, which allows for clear visualization of methylation differences during iPSC reprogramming (relative to MEFs). This approach validates the targeted demethylation findings in both female and male cells (Figures S3A and S3B), and more importantly it allows for statistical significance to be calculated after grouping of the differences elicited by reprogramming, irrespective of the differences in the process or the genome coverage and sequencing depth of the data. Based on these analyses, we observed that DNA demethylation at ESC enhancers and super-enhancers is already evident in the intermediate-late stages of reprogramming but becomes yet more pronounced in d60 iPSCs (Figures 3B, 3C, S3C, and S3D). Notably, we identified targeted demethylation at these same regulatory regions in pre-implantation embryos and during the serum to 2i transition (Figure 3D) while it was absent in unipotent PGCs (Surani, 2012). These results reveal a conserved targeted demethylation signature during reprogramming to pluripotent cell identity in vitro and in vivo, which seems to be independent of the extent of global DNA demethylation.

### AID Regulates UHRF1 and Global Demethylation during iPSC Reprogramming

**Figure 4:**
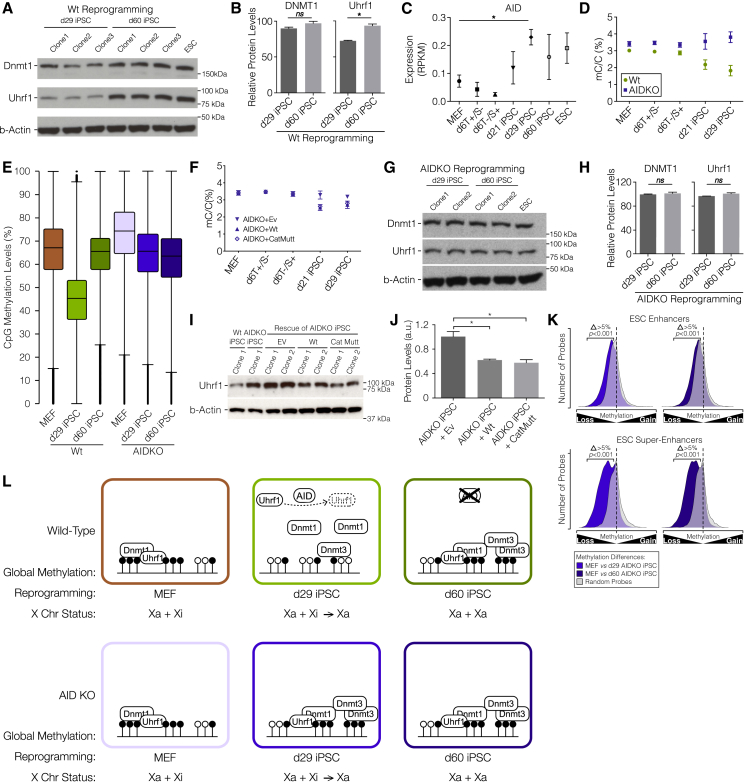
Mechanisms of DNA Demethylation (A) WB showing levels of DNMT1 and UHRF1 in WT iPSCs at d29 and d60. β-actin was used as a loading control. (B) Quantitation of DNMT1 and UHRF1 WB bands, relative to ESCs levels. (C) Expression profile (RPKM) for *Aid*. p values shown are the result of two-tailed t tests from MEFs to d29 iPSCs. (D) Global 5mC levels, measured by LC-MS. Results are expressed as percentage of total cytosine. Data are represented as mean ± SEM. Shown are results for reprogramming of WT and AidKO MEFs. (E) Global CpG methylation levels, as assessed by BS-seq, for every time point during AidKO MEF reprogramming. Plot displays the median (bar), inter-quartile range (box), and maximum and minimum (whiskers). (F) Global 5mC levels, measured by LC-MS. Results are expressed as percentage of total cytosine. Data are represented as mean ± SEM. Shown are results for AidKO MEFs reprogrammed with the OSKM plus an empty vector (EV), a vector containing AID WT cDNA (AIDWt), or a vector containing AID catalytic mutant cDNA (AIDCatMutt). (G) WB showing levels of DNMT1 and UHRF1 in AidKO iPSCs at d29 and d60. β-actin was used as a loading control. (H) Quantitation of DNMT1 and UHRF1 WB bands, relative to ESCs levels. (I) WB showing levels of UHRF1 in WT and AidKO iPSCs d29 and clones rescued with EV, AIDWtor AIDCatMutt. β-actin was used as a loading control. (J) Quantitation of UHRF1 WB bands (arbitrary units [a.u.]). (K) Density plots of methylation differences at ESC enhancer and SE from AidKO MEFs to iPSCs, shown by opaque plots. Overlaid by MMRP transparent gray density plot. Δ denotes a minimum 5% difference between data and MMRP profile p values shown are the result of a pairwise t test with a Benjamini-Hochberg correction. (L) Proposed model to explain role of AID in DNA methylation dynamics during iPSC reprogramming. See also Figure S4.

To understand the molecular regulation of global demethylation associated with female cell reprograming, we assessed the expression levels of genes implicated in DNA methylation and demethylation. As previously shown (Buganim et al., 2012, Polo et al., 2012), all *Dnmt* genes were significantly upregulated upon reprogramming (Figure S4A). Importantly, we also confirmed upregulation of DNMT1 and DNMT3b proteins (Figures 4A and S4B). However, we observed one notable exception to this pattern UHRF1, responsible for the recruitment of DNMT1 to hemi-methylated DNA (Bostick et al., 2007, Sharif et al., 2007). While its transcription was upregulated by reprogramming, we observed substantially reduced protein levels at the stages associated with global hypomethylation (Figures 4A and 4B), when compared to fully reprogrammed iPSCs.

AID has been previously implicated in iPSC reprogramming, but the timing, mechanisms, and extent of demethylation it may regulate are unknown (Bhutani et al., 2013, Kumar et al., 2013). It was therefore interesting to note that *Aid* expression peaked precisely in d29 iPSCs (Figure 4C) when DNA methylation levels are lowest. Moreover, global demethylation during reprogramming of female Aid knock-out (AidKO) MEFs was much less substantial and was delayed when compared to wild-type (WT) female cell reprogramming (Figures 4D, 4E, S4C, and S4D). Typical reprograming-induced demethylation in female cells was partially rescued by re-expression of either the wild-type or a catalytically mutant isoform of AID, but not by expression of an empty vector (Figure 4F). These results show the importance of AID in regulating global DNA demethylation during reprogramming and that this regulation is independent of the deaminase activity of AID.

Given the marked abrogation of demethylation observed during reprograming of female AidKO MEFs, and the downregulation of UHRF1 protein associated with reprograming and global hypomethylation in WT cells, we compared UHRF1 protein levels during WT versus AidKO cell reprograming. Intriguingly, deficiency in AID prevented the downregulation of UHRF1 protein (Figures 4G and 4H). In addition, overexpression of AID (both WT and deaminase mutant) in AidKO reprogramming cells led to a significant decrease in UHRF1 protein levels (Figures 4I and 4J), consistent with a role for AID in regulating its abundance at a posttranscriptional level and reinforcing its importance in regulating global demethylation. We note in this respect the differential expression of genes involved in ubiquitination (which is known to regulate UHRF1 [Chen et al., 2013a]) between WT and AidKO d29 iPSCs (Figure S4E), which included *Lonrf3*, *Mdm2*, *Usp48*, *Pramel7*, *Rnf32*, *Shprh*, and *Trim17* among others.

It is notable that despite the profound defect in the transient global demethylation associated with reprograming, we detected no differences in global methylation levels between WT and AidKO d60 iPSCs, presumably due to the de novo methylation wave that takes place at the later stages of reprograming. However, targeted demethylation at ESC-specific enhancers and super-enhancers was not affected by lack of AID (Figure 4K) consistent with the fact that in general activation of the pluripotency transcriptional program occurred normally in AidKO iPSCs (Figure S4F). This is consistent with the mild effects of AID deficiency on obtaining iPSCs (Habib et al., 2014, Shimamoto et al., 2014). However we did identify more than 17,000 DMRs in AidKO iPSCs, most of which (72%) were hypermethylated (Figure S4G). These hypermethylated DMRs occur throughout the genome and in all genomic features, consistent with a global effect of AID. Additionally, these cells appear to have impaired differentiation potential, as they are unable to upregulate several differentiation markers at the same levels as WT cells (Figure S4H). These findings reconcile previous observations on AidKO iPSCs (Kumar et al., 2013), showing that global demethylation is mechanistically uncoupled from targeted demethylation and is necessary for the erasure of epigenetic memory.

Our data also showed significant upregulation of the ten-eleven Translocation (Tet) *Tet1* and *Tet2,* and *Tdg* genes in d29 iPSCs (Figure S4I) that continue to be highly expressed in fully reprogrammed iPSCs. Hydroxymethylation levels were low in MEFs and d6T^+^/S^–^ cells, in contrast to d6T^–^/S^+^ cells that have hydroxymethylation levels similar to primed ESCs (Figure S4J). This increase in 5-hydroxymethylcytosine (5hmC) correlates with the observed increase in expression of the *Tet* enzymes, consistent with their role in controlling MET (Hu et al., 2014). Furthermore, the expression dynamics of *Tet1*, *2* and *Tdg* are consistent with a possible role in targeted but not in global demethylation.

Finally, based on the observation that female and male cells undergo global DNA demethylation to different extents, and when female cells are devoid of AID they show an extent of global demethylation similar to male WT cells, we investigated the status of X chromosome reactivation. We observed that AidKO female cells are able to reactivate the X chromosome, just like WT cells (Figure S4K). Furthermore, to exclude that this could be a reprogramming system-specific effect, we reprogrammed fibroblasts from one male and four female human donors. Human cells also undergo global demethylation during reprogramming (Figure S4L). However, this demethylation is not as profound as in female mouse cells, resembling more closely the male mouse global DNA methylation profile. Since human cells do not robustly reactivate the X chromosome during iPSC reprogramming (Tchieu et al., 2010), we investigated whether this was the case in our reprogramming system. We observed that our human cells were not able to reactivate the X chromosome and *Xist* expression was maintained (Figures S4M and S4N). Moreover, we observed that *Aid* is expressed at d11, when methylation levels are lower (Figure S4O).

These results suggest that global DNA demethylation during reprogramming is mainly achieved by passive demethylation, similar to what has been reported in other reprogramming processes (Seisenberger et al., 2012). Furthermore, AID can influence the global methylation levels during reprograming by regulating the protein levels of UHRF1, and thus the efficiency of recruitment of the maintenance methylation machinery. Additionally, the extent of global DNA demethylation is not dependent on the reprogramming system or species but seems to be influenced by the capacity of cells to reactivate the X chromosome (Figure 4L).

## Discussion

The extent and role of DNA methylation remodeling during the reprogramming of somatic cells to pluripotency are poorly understood. Our detailed and comprehensive study reveals that iPSCs undergo transient global demethylation during reprogramming and that stable targeted demethylation occurs in parallel to the global one. Notably, we show that the targeted and global demethylation processes are mechanistically uncoupled and that upregulation of pluripotency genes precedes and is not dependent on the extent of global demethylation. Targeted demethylation establishes a unique epigenetic pluripotency signature, which is broadly conserved in other reprogramming processes. An important caveat is that there are gender-specific differences in the extent to which the genome demethylates globally. In female cells, where DNA demethylation is more pronounced, downregulation of UHRF1 protein, through an AID-dependent mechanism, facilitates global but not targeted demethylation (Figure 4L). Moreover, our results clearly show that cells lacking AID-mediated global demethylation have an impaired differentiation potential, showing that AID is important for epigenetic memory erasure but not for the establishment of pluripotent cell identity.

Global DNA demethylation occurs in early embryos, during PGC development and in naive ESCs in both mouse and human (von Meyenn and Reik, 2015) and has consequently been proposed to be a conserved and obligate feature of reprogramming (Lee et al., 2014b, Nashun et al., 2015). Here, we show that mouse female and male cells undergo different levels of genome-wide demethylation during iPSC reprogramming. Our results, extrapolated from human cell reprogramming, point to a role for X chromosome reactivation in influencing these differences. This is consistent with mouse female ESCs having lower global methylation levels than male ones (Zvetkova et al., 2005) and with a recent report in PGC-like cell induction, where female cells undergo DNA methylation reprogramming similar to male cells, however, with more pronounced global changes (Shirane et al., 2016).

We reprogram MEFs to iPSCs in the presence of serum, which in ESCs results in high global methylation levels similar to those of somatic cells (Ficz et al., 2013, Habibi et al., 2013, Leitch et al., 2013). Hence despite high levels of de novo methyltransferases, controlled downregulation of UHRF1 protein seems critical for global demethylation. We have recently shown that UHRF1 is also regulated at the protein level when mouse ESCs are transitioned from serum to 2i (von Meyenn et al., 2016). This potentially provides a unifying theme for genome-wide demethylation mechanisms, which in mice and humans are characterized by disabling of the UHRF1/DNMT1 system, including by posttranscriptional regulation of *Uhrf1* (Seisenberger et al., 2012, Sugawa et al., 2015).

The role of AID in DNA demethylation and reprogramming in vivo and in vitro has been puzzling with the majority of studies demonstrating that it plays a role in demethylation (Bhutani et al., 2010, Bhutani et al., 2013, Kumar et al., 2013, Popp et al., 2010, Santos et al., 2013) but that it has mild impact on iPSC reprogramming (Habib et al., 2014, Shimamoto et al., 2014). Our results clearly show that AID plays a major role in global DNA demethylation, and unexpectedly this seems to be brought about by its negative regulation of UHRF1 protein levels, suggesting a novel role for AID in posttranslational regulation of UHRF1. Known mechanisms of UHRF1 regulation that can affect DNA methylation include ubiquitination among others (Tauber and Fischle, 2015). We note in this respect that several ubiquitination and deubiquitination enzymes are differentially expressed in iPSCs with and without AID, and that AID itself interacts with a ubiquitin ligase (Sun et al., 2013).

Targeted demethylation (over and above the global demethylation) occurs at ESC-specific enhancers and super-enhancers to a similar extent in female and male cells, and this is conserved in other reprogramming processes in which pluripotent cell identity is achieved. These regions share the characteristics of being CpG-poor and transcription factor (TF) binding-rich regions, characteristics that have been proposed to play a role in focal or targeted demethylation (Soufi et al., 2012, Stadler et al., 2011). TET proteins have also been implicated in reprogramming (Nashun et al., 2015), as well as in targeted enhancer (Pastor et al., 2013) and super-enhancer (Ding et al., 2015) demethylation in ESCs. However, Tet enzymes are needed specifically for activation of microRNAs essential for iPSC derivation, and it is possible to obtain fully reprogrammed iPSCs from *Tet1-3* triple-knockout MEFs after ectopic expression of miR200c (Hu et al., 2014). Nevertheless, the dynamics of *Tet* enzyme expression and of the hydroxymethylation levels that we observe suggest they may play a role in the fine-tuning of targeted demethylation. Indeed, a model that seeks to explain DNA methylation dynamics at enhancers during differentiation has been proposed (Hon et al., 2014). This model suggests that at TF binding-rich enhancers, binding of TFs excludes DNMT1 activity, leading to their demethylation, whereas in TF binding-poor enhancers, TET2 protein is crucial in fine-tuning enhancer methylation in an oxidation-dependent manner. We suggest that a similar mechanism could be responsible for the targeted remodeling of these regions during reprogramming.

In contrast, AID does not have a role in targeted demethylation of pluripotency regulatory regions, but its absence results in widespread hypermethylated epialleles in iPSCs. This explains why AID-deficient iPSCs can be obtained, but cells with residual and persistent epigenetic memory may well behave aberrantly and unpredictably in potential future therapeutic settings, given their altered differentiation potential. It will be interesting to investigate further the differences in developmental potential between female and male iPSCs, which could impact on their use in basic and translational research. Hence, in a process where cells have to switch off a somatic expression program and upregulate a pluripotency network, global DNA demethylation seems to be important for the removal of epigenetic memory, while targeted demethylation at regulatory regions, and, in particular, at ESC super-enhancers, is crucial for the establishment of the pluripotent identity. Understanding and manipulating the two demethylation processes may result in improvements in the safety and the efficiency of obtaining robust, high quality iPSCs, prerequisites for therapeutic applications in regenerative medicine.

## Experimental Procedures

### Reprogramming of MEFs to iPSCs

For each transfection, 0.8 × 10^6^ MEFs were nucleofected using Amaxa Nucleofection Technology (Lonza AG; program A-023), according to the manufacturer’s instructions, with 1 μg of each plasmid. Plasmids for reprogramming pB-TRE-OCKS, pBASE, and pB-CAG-rtTA were obtained from the Wellcome Trust Sanger Institute’s plasmid repository. Reprogramming was performed in ESC medium (DMEM, 15% fetal bovine serum, 1% anti-anti, 1% MEM non-essential amino acids, 50 μM b-mercaptoethanol, and 10^3^ U leukemia inhibitory factor [LIF]) in the presence or absence of doxycycline, in a 5% O_2_ incubator. The medium was refreshed every other day. Colonies were picked on day 6 of reprogramming and expanded for at least 54 days. Cells were collected at different time points during reprogramming: mouse embryonic fibroblasts (MEFs), d6 fluorescence-activated cell sorted (FACS) refractory cells positive for Thy1 and negative for SSEA1 surface markers (d6T^+^/S^–^), and early reprogramming intermediates negative for Thy1 and positive for SSEA1 (d6T^–^/S^+^) as well as the reprogramming of individual colonies at intermediate-late stages of reprogramming (d21 iPSC and d29 iPSC) and established iPSCs (d60 iPSC).

All animal work carried out in this study is covered by a project license under the Animal (Scientific Procedures) Act 1986, and further regulated by the Babraham Institute Animal Welfare, Experimentation, and Ethics Committee.

## Author Contributions

I.M. conceived the project, designed and performed experiments, analyzed data, and wrote the manuscript; T.M.S. performed all PBAT libraries and data processing and provided helpful discussions; M.R.K. and J.S. performed WB experiments and karyotyping and provided technical assistance and helpful discussions; F.S. performed all immunofluorescence (IF) experiments; M.B. and S.B. performed all mass spectrometry experiments; F.K. and S.R.A. provided bioinformatics support; A.S.-P. performed all statistical analysis; W.D. performed all embryo work and provided helpful discussions for project design, and W.R. interpreted the data, provided helpful discussions for project design, and helped write the manuscript. All authors have interpreted the data and provided helpful discussions and read and approved the manuscript.

## Accession Numbers

The accession number for the BS-seq and RNA-seq data reported in this paper is GEO: GSE69823.

## Supplemental Information

Document S1. Supplemental Experimental Procedures and Figures S1–S4

Document S2. Article plus Supplemental Information
