# Supplementary material for: Gender Differences in Global but Not Targeted Demethylation in iPSC Reprogramming
Source: Cell Rep. 2017 Jan 31;18(5):1079–89. doi: 10.1016/j.celrep.2017.01.008 (PMC5300890; doi:10.1016/j.celrep.2017.01.008)
Supplement: Document S1. Supplemental Experimental Procedures and Figures S1–S4 [file mmc1.pdf]

Cell Reports, Volume *18*

Supplemental Information

Gender Differences in Global but Not Targeted Demethylation in iPSC Reprogramming

Inês Milagre, Thomas M. Stubbs, Michelle R. King, Julia Spindel, Fátima Santos, Felix Krueger, Martin Bachman, Anne Segonds-Pichon, Shankar Balasubramanian, Simon R. Andrews, Wendy Dean, and Wolf Reik

Figure S1

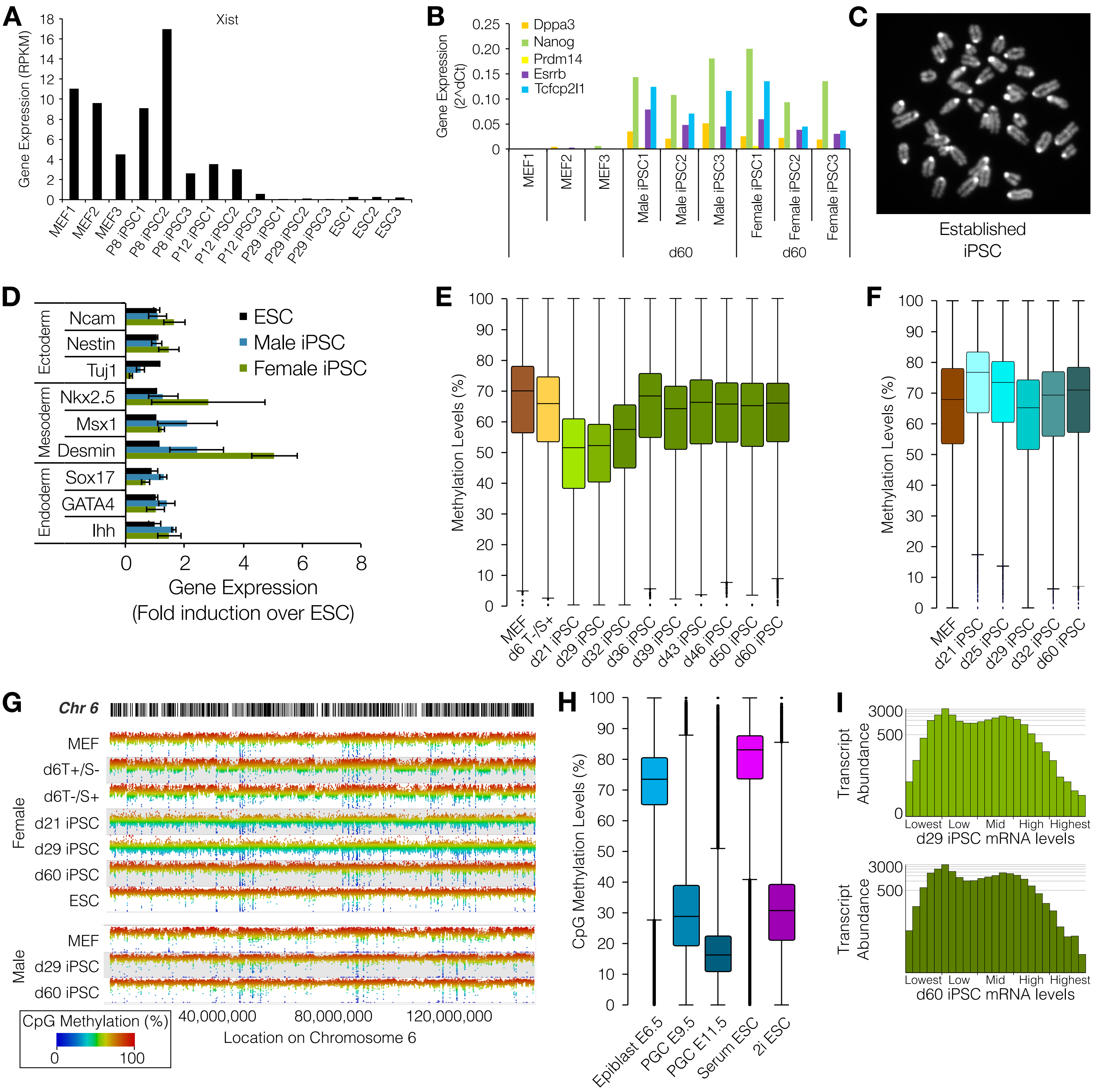

A
Xist
Gene Expression (RPKM)
MEF1
MEF2
MEF3
P8 iPSC1
P8 iPSC2
P8 iPSC3
P12 iPSC1
P12 iPSC2
P12 iPSC3
P29 iPSC1
P29 iPSC2
P29 iPSC3
ESC1
ESC2
ESC3
B
Dppa3
Nanog
Prdm14
Esrrb
Tcfcp2l1
Gene Expression (2^dCt)
MEF1
MEF2
MEF3
Male iPSC1
Male iPSC2
Male iPSC3
Female iPSC1
Female iPSC2
Female iPSC3
d60
d60
C
Established iPSC
D
Ectoderm
Mesoderm
Endoderm
Ncam
Nestin
Tuj1
Nkx2.5
Msx1
Desmin
Sox17
GATA4
Ihh
ESC
Male iPSC
Female iPSC
Gene Expression (Fold induction over ESC)
E
Methylation Levels (%)
MEF
d6 T-/S+
d21 iPSC
d29 iPSC
d32 iPSC
d36 iPSC
d39 iPSC
d43 iPSC
d46 iPSC
d50 iPSC
d60 iPSC
F
Methylation Levels (%)
MEF
d21 iPSC
d25 iPSC
d29 iPSC
d32 iPSC
d60 iPSC
G
Chr 6
Female
MEF
d6T+/S-
d6T-/S+
d21 iPSC
d29 iPSC
d60 iPSC
ESC
Male
MEF
d29 iPSC
d60 iPSC
CpG Methylation (%)
0
100
40,000,000
80,000,000
120,000,000
Location on Chromosome 6
H
CpG Methylation Levels (%)
Epiblast E6.5
PGC E9.5
PGC E11.5
Serum ESC
2i ESC
I
Transcript Abundance
Lowest Low Mid High Highest
d29 iPSC mRNA levels
Transcript Abundance
Lowest Low Mid High Highest
d60 iPSC mRNA levels

Figure S1: iPSC reprogramming system and global DNA methylation (Related to Figure 1) (A) Expression profile (in RPKM) of *Xist* in each female iPSC clone and comparison with ESCs. (B) Expression profile (qPCR) of pluripotency genes upregulated in each iPSC clone and comparison with MEFs. (C) Representative karyotype of an established iPSC clone. (D) Differentiation markers expressed in embryoid bodies (EB) from each iPSC clone and comparison with EB from ESCs. (E-F) CpG methylation levels, as assessed by PBAT BS-Seq, at additional time-points of reprogramming of (E) female and (F) male cells. Plot displays the median (bar), inter-quartile range (box), and maximum and minimum (whiskers). (G) Methylation distribution and levels of each 50 CpG probe across chromosome 6, for all time-points during female and male cell reprogramming. (H) CpG methylation levels, as assessed by BS-Seq, for PGC development (Epiblast, PGCs E9.5 and E11.5) and ESCs (in serum and 2i conditions). Plot displays the median (bar), inter-quartile range (box), and maximum and minimum (whiskers). (I) Histogram of transcript abundance for genes showing different expression levels at d29 and d60 iPSCs.

Figure S2

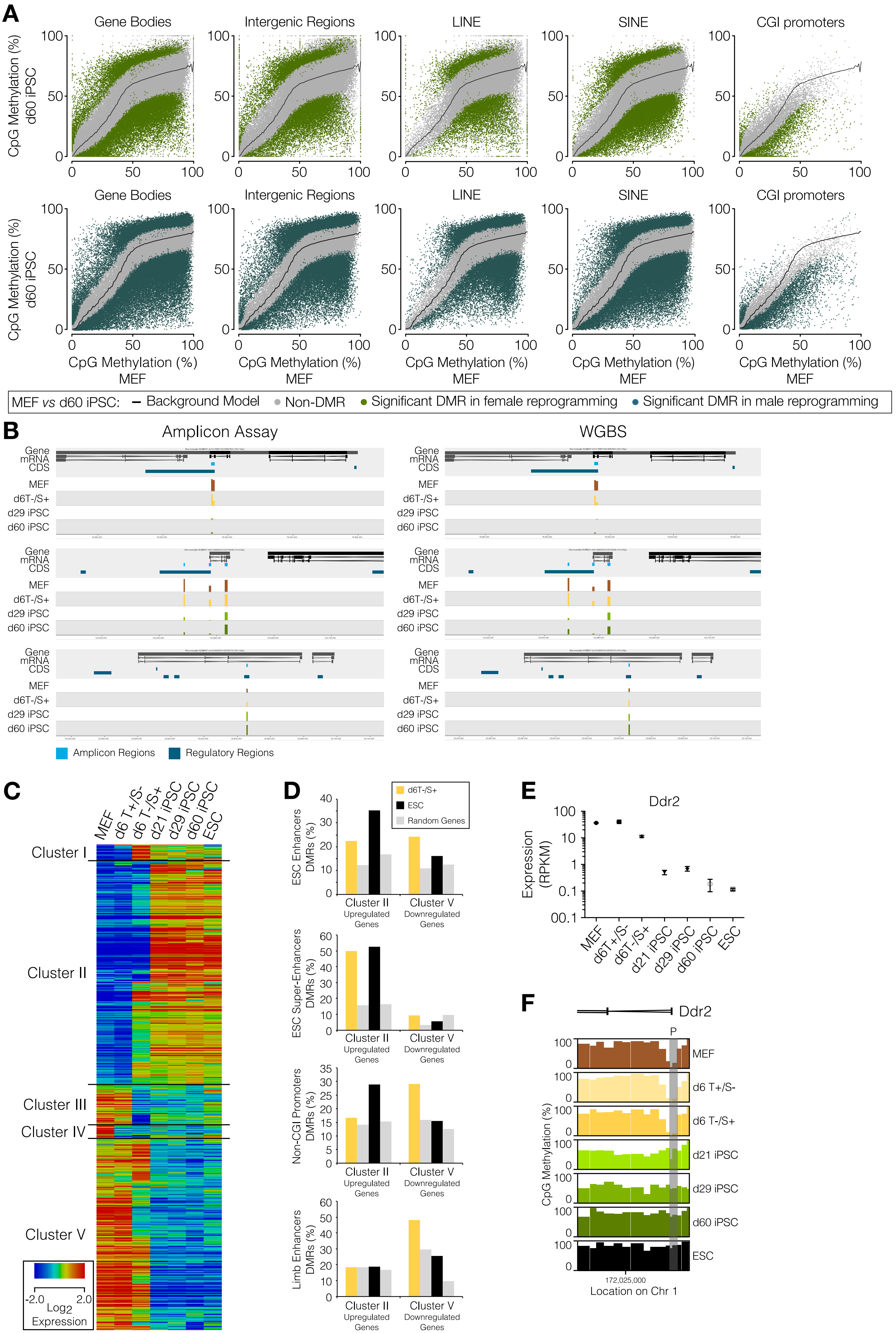
A
Gene Bodies
Intergenic Regions
LINE
SINE
CGI promoters
CpG Methylation (%) d60 iPSC
CpG Methylation (%) MEF
MEF vs d60 iPSC: Background Model Non-DMR Significant DMR in female reprogramming Significant DMR in male reprogramming
B
Amplicon Assay
WGBS
Gene
mRNA
CDS
MEF
d6T-/S+
d29 iPSC
d60 iPSC
Amplicon Regions
Regulatory Regions
C
MEF
d6 T+/S-
d6 T-/S+
d21 iPSC
d29 iPSC
d60 iPSC
ESC
Cluster I
Cluster II
Cluster III
Cluster IV
Cluster V
-2.0
2.0
Log2 Expression
D
d6T-/S+
ESC
Random Genes
ESC Enhancers DMRs (%)
ESC Super-Enhancers DMRs (%)
Non-CGI Promoters DMRs (%)
Limb Enhancers DMRs (%)
Cluster II Upregulated Genes
Cluster V Downregulated Genes
E
Ddr2
Expression (RPKM)
MEF
d6T+/S-
d6T-/S+
d21 iPSC
d29 iPSC
d60 iPSC
ESC
F
Ddr2
P
CpG Methylation (%)
MEF
d6 T+/S-
d6 T-/S+
d21 iPSC
d29 iPSC
d60 iPSC
ESC
172,025,000
Location on Chr 1

Figure S2: Relationship between DNA methylation and gene expression (Related to Figure 2)

(A) Scatter plot of DNA methylation levels of individual probes genome-wide, showing different genomic features of MEFs and d60 iPSCs. Dots represent individual 50 CpG probes – significant differentially methylated regions (DMRs) are represented in green (female cell reprogramming) or blue (male cell reprogramming). Background model depicted as a black line. (B) Methylation profile for all regions confirmed by Amplicon-BS-Seq assay and comparison to WGBS profile. (C) Differentially expressed gene expression heat map. Clusters I to V represent different expression patterns during reprogramming. (D) Percentage of DMRs at regulatory regions, overlapping specific gene clusters. Shown are DMRs containing ESC enhancers and super-enhancers, non-CGI promoters and limb enhancers and their overlap with genes that are up-regulated during reprogramming (Cluster II) and genes that are down-regulated during reprogramming (Cluster V), comparing to random sets with the same number of genes, for d6T-/S+ and ESCs. (E) Expression profile (RPKM) of *Ddr2* gene. (F) Example of BS-seq profile for MEF gene *Ddr2* promoter region. Methylation levels of individual probes, between 0 and 100% are shown. Shadowed areas highlight promoter – P.

Figure S3

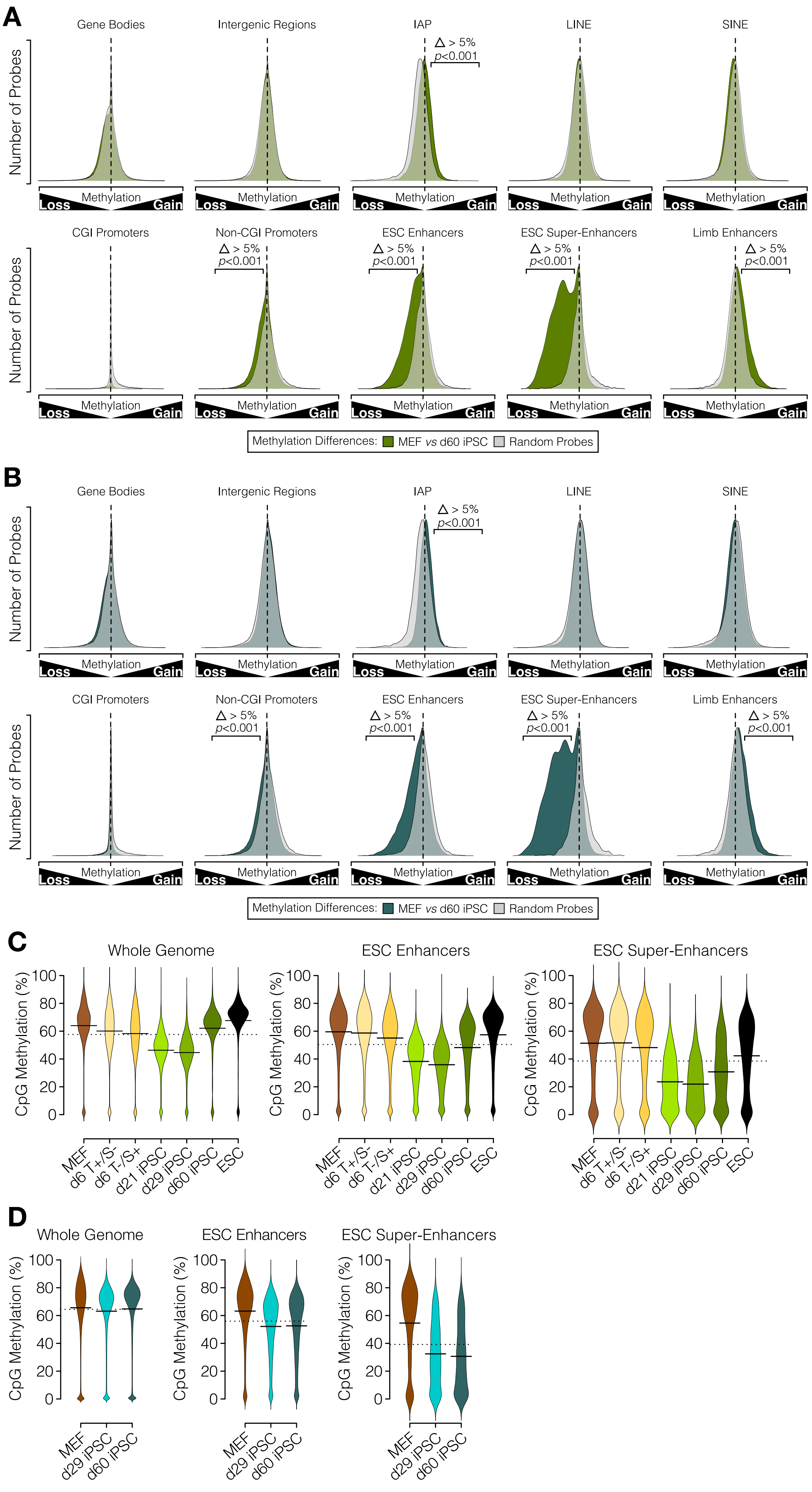
A
Gene Bodies
Intergenic Regions
IAP
LINE
SINE
Number of Probes
Δ > 5%
p<0.001
Methylation
Loss
Gain
CGI Promoters
Non-CGI Promoters
ESC Enhancers
ESC Super-Enhancers
Limb Enhancers
Methylation Differences: MEF vs d60 iPSC Random Probes
B
Gene Bodies
Intergenic Regions
IAP
LINE
SINE
CGI Promoters
Non-CGI Promoters
ESC Enhancers
ESC Super-Enhancers
Limb Enhancers
Methylation Differences: MEF vs d60 iPSC Random Probes
C
Whole Genome
ESC Enhancers
ESC Super-Enhancers
CpG Methylation (%)
0
20
40
60
80
100
MEF
d6 T+/S-
d6 T-/S+
d21 iPSC
d29 iPSC
d60 iPSC
ESC
D
Whole Genome
ESC Enhancers
ESC Super-Enhancers
CpG Methylation (%)
MEF
d29 iPSC
d60 iPSC

Figure S3: DNA demethylation dynamics at different genomic features (Related to Figure 3)

(A-B) Density plots of methylation differences for different genomic features from MEFs to d60 iPSCs, shown by opaque dark green (A-female) or dark blue (B-male) plots. Overlaid by MMRP transparent grey density plot. Δ denotes a minimum 5% difference between data and MMRP profile. *p*-values shown are the result of a pairwise *t*-test with a Benjamini-Hochberg correction. (C-D) CpG methylation levels for whole genome, ESC enhancers and super-enhancers, for female (C) and male (D) reprogramming. Beanplots show the density of loci for each methylation value.

Figure S4

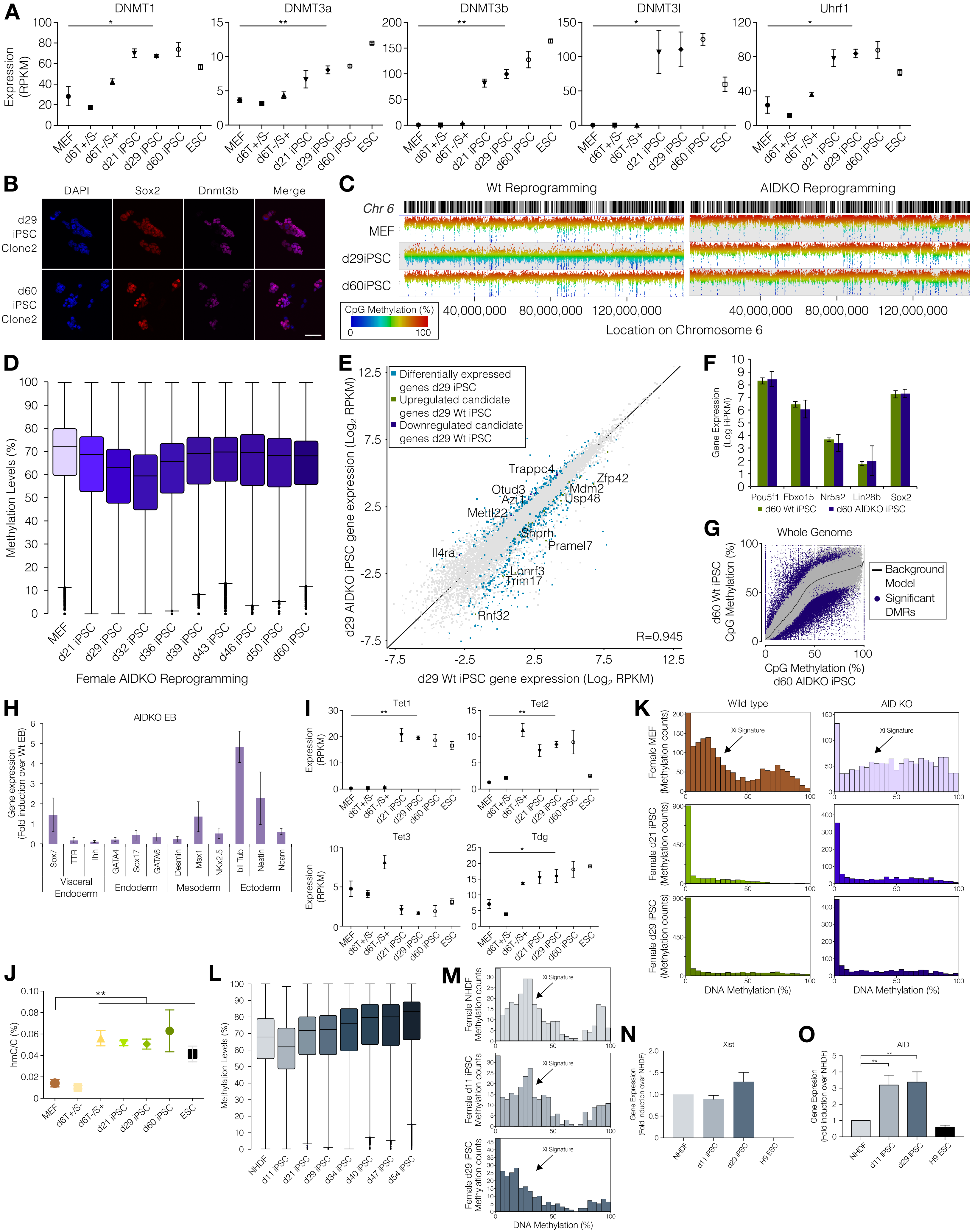
A
DNMT1
DNMT3a
DNMT3b
DNMT3l
Uhrf1
Expression (RPKM)
MEF
d6T+/S-
d6T-/S+
d21 iPSC
d29 iPSC
d60 iPSC
ESC
B
DAPI
Sox2
Dnmt3b
Merge
d29 iPSC Clone2
d60 iPSC Clone2
C
Wt Reprogramming
AIDKO Reprogramming
Chr 6
MEF
d29iPSC
d60iPSC
CpG Methylation (%)
40,000,000
80,000,000
120,000,000
Location on Chromosome 6
D
Methylation Levels (%)
MEF
d21 iPSC
d29 iPSC
d32 iPSC
d36 iPSC
d39 iPSC
d43 iPSC
d46 iPSC
d50 iPSC
d60 iPSC
Female AIDKO Reprogramming
E
Differentially expressed genes d29 iPSC
Upregulated candidate genes d29 Wt iPSC
Downregulated candidate genes d29 Wt iPSC
d29 AIDKO iPSC gene expression (Log2 RPKM)
d29 Wt iPSC gene expression (Log2 RPKM)
Trappc4
Zfp42
Otud3
Mdm2
Azi1
Usp48
Mettl22
Shprh
Pramel7
Il4ra
Lonrf3
Trim17
Rnf32
R=0.945
F
Gene Expression (Log RPKM)
Pou5f1
Fbxo15
Nr5a2
Lin28b
Sox2
d60 Wt iPSC
d60 AIDKO iPSC
G
Whole Genome
d60 Wt iPSC CpG Methylation (%)
CpG Methylation (%) d60 AIDKO iPSC
Background Model
Significant DMRs
H
AIDKO EB
Gene expression (Fold induction over Wt EB)
Sox7
TTR
Ihh
GATA4
Sox17
GATA6
Desmin
Msx1
NKx2.5
bIIITub
Nestin
Ncam
Visceral Endoderm
Endoderm
Mesoderm
Ectoderm
I
Tet1
Tet2
Tet3
Tdg
Expression (RPKM)
K
Wild-type
AID KO
Female MEF (Methylation counts)
Female d21 iPSC (Methylation counts)
Female d29 iPSC (Methylation counts)
Xi Signature
DNA Methylation (%)
J
hmC/C (%)
L
Methylation Levels (%)
NHDF
d11 iPSC
d21 iPSC
d29 iPSC
d34 iPSC
d40 iPSC
d47 iPSC
d54 iPSC
M
Female NHDF Methylation counts
Female d11 iPSC Methylation counts
Female d29 iPSC Methylation counts
Xi Signature
DNA Methylation (%)
N
Xist
Gene Expression (Fold induction over NHDF)
NHDF
d11 iPSC
d29 iPSC
H9 ESC
O
AID
Gene Expresion (Fold induction over NHDF)

Figure S4: Mechanisms and global DNA demethylation in different reprogramming systems (Related to Figure 4)

(A) Expression profile (in RPKM) of *Dnmt1, 3a, 3b, 3l* and *Uhrf1*. *p*-values shown are the result of two-tailed *t*-tests from MEFs to d29 iPSCs. Data are represented as mean ± SEM. (B) Immunofluorescence (IF) of staining of Wt iPSCs at d29 and d60. Shown are representative IF for DAPI, SOX2 and DNMT3b for one iPSC clone. (C) Methylation distribution and levels of each 50 CpG probe across chromosome 6, for MEFs, d29 and d60 iPSCs during AIDKO MEF reprogramming. (D) CpG methylation levels, as assessed by PBAT BS-Seq, at additional time-points of reprogramming of AIDKO cells. Plot displays the median (bar), inter-quartile range (box), and maximum and minimum (whiskers). (E) Scatter plot of RNA expression levels in d29 Wt and AIKO iPSCs. Differentially expressed genes (blue), upregulated (green) and downregulated (purple) candidate genes. (F) Expression profile (RPKM) of pluripotency genes in Wt and AIDKO d60 iPSCs. (G) Scatter plot of DNA methylation levels of individual probes genome-wide, showing whole genome of d60 Wt and AIDKO iPSCs. Individual dots represent individual 50 CpG probes, DMRs are represented in purple and probes that do not differ between samples in grey. Background model depicted as a black line. (H) Differentiation markers expressed in embryoid bodies (EB) from AIDKO iPSCs, shown are fold induction values compared to Wt iPSCs. (I) Expression profile (in RPKM) of *Tet1-3* and *Tdg*. *p*-values shown are the result of two-tailed *t*-tests from MEF to d29 iPSCs. Data are represented as mean ± SEM. (J) Global 5hmC levels, measured by LC-MS. Results are expressed as percentage of total cytosine. Data are represented as mean ± SEM. *p*-values shown are the result of an ANOVA multiple-comparison test with Dunnett's multiple comparison test: $p<0.0001$. (K) Distribution of methylation levels across CpG islands on the X chromosome in indicated cell types and time-points during reprogramming, based on BS-Seq data. The arrow indicates the Xi-specific DNA methylation signature. (L) CpG methylation levels, as assessed by PBAT BS-Seq, during reprogramming of human cells. Plot displays the median (bar), inter-quartile range (box), and maximum and minimum (whiskers). (M) Distribution of methylation levels across CpG islands on the X chromosome in indicated cell types during human iPSC reprogramming, based on PBAT BS-Seq data. The arrow indicates the Xi-specific DNA methylation signature. (N-O) Expression profile (qPCR) of (N) *Xist* and (O) *Aid* during human female reprogramming. Data are represented as mean ± SEM. *p*-values shown are the result of an ANOVA multiple-comparison test with Dunnett's multiple comparison test: $p<0.01$.

Supplemental Experimental Procedures

Cell culture

ESC and iPSC culture – TMBD10 ESCs (derived by the gene targeting facility at the Babraham Institute from C57Bl/6 mice) and iPSCs were grown in ESCs medium (DMEM, 15% fetal bovine serum, 1% Anti-Anti, 1% MEM Non-Essential Amino Acids, 50 µM 2-mercaptoethanol and 10^3 U LIF). ES and iPS cells were grown either in complete ESC medium in gelatinized dishes or differentiated for 11 days into embryoid bodies via LIF removal and suspension culture.

Isolation of MEF - 13.5 to 14.5 days post coitum (d.p.c.) Wild-type Oct4-GFP or AidKO Oct4-GFP embryos were decapitated, eviscerated, dissociated with 0.25% trypsin, 0.1% EDTA and plated onto MEF medium (DMEM, 10% FBS, 1% Anti-Anti, 1% MEM Non-Essential Amino Acids and 50 µM 2-mercaptoethanol). The Oct4/GFP strain carries a transgene comprising 18kb of the endogenous murine Oct4 locus including downstream regulatory elements and the endogenous Oct4 promoter from which green fluorescent protein (GFP) is expressed (Yeom et al., 1996; Yoshimizu et al., 1999), the AidKO Oct4-GFP has been previously described and characterized (Popp et al., 2010). All animal work carried out in this study is covered by a project license under the Animal (Scientific Procedures) Act 1986, and further regulated by the Babraham Institute Animal Welfare, Experimentation, and Ethics Committee.

Rescue experiments – For each transfection, $0.8x10^6$ MEFs were nucleofected with reprogramming plasmids plus an empty vector, Wt or Catalytic-mutant AID. Cells were selected with blasticidin for 5 days. Picked and expanded colonies were collected at d21 and d29 and DNA extracted to be used in LC-MS analysis and protein extracts used in WB.

Reprogramming of human Fibroblasts to iPSCs

Reprogramming was performed by infecting $3.0x10^5$ normal human dermal fibroblasts (NHDF - GIBCO) with CytoTune®-iPS 2.0 Sendai Reprogramming Kit (Invitrogen), according to manufacturer's instruction, at an MOI of 1. Cells were maintained in fibroblast medium (DMEM, 10% FBS, 1% Anti-Anti, 1% MEM Non-Essential Amino Acids and 50 µM 2-mercaptoethanol) for five days. Infected cells were then replated onto feeder dishes and maintained in KSR medium (Advanced DMEM:F12, 20% KSR, 1% Anti-Anti, 1% MEM Non-Essential Amino Acids, 25 µM 2-mercaptoethanol and 4ng/ml bFGF). Medium was replenished everyday. Cells were collected at different time-points during reprogramming by FACS (NHDF, d11, d21, d29) or manually (d34, d40, d47 and d54).

Fluorescence-activated cell sorting (FACS)

For cell sorting, mouse cells were harvested and incubated with antibodies against Thy1.2 (PE, eBioscience) and SSEA-1 (eFluor 660, eBioscience), human cells were incubated with antibodies against CD13 (PE, BD Pharmigen) and SSEA-4 (Alexa Fluor 647, BD Pharmigen) for 30 min. Cells were washed in a 2% FBS/PBS solution and passed through a 50µm cell strainer to achieve single-cell suspension. Cells were stained with DAPI just prior to sorting. Appropriate negative and positive controls were used to assess optimal FACS conditions. Cell sorting was performed using an Influx cell sorter instrument (BD Biosciences) and cells were collected for downstream applications.

Karyotyping

Cells were grown in gelatinised dishes overnight and colchicine (0.04 μg/ml) was added to the medium for 1 hour. Subsequently, cells were trypsinised to single cells, pelleted and resuspended in hypotonic buffer KCl 0.075M. After 5-minute incubation at 37ºC, cells were fixed in ice-cold acetic/methanol 1:3 at 4ºC for 2 hours. Cells were dropped onto chilled slides, stained with DAPI for 2 minutes and observed under an Olympus BX61 fluorescence microscope. At least 80 chromosome spreads were counted and each cell line was classified as having normal or abnormal karyotypes.

Liquid chromatography – Mass-spectrometry
Genomic DNA (a minimum of 100ng) was incubated with DNA Degradase Plus (Zymo Research) at 37°C for 4 h. Detection of global mC and hmC levels was carried out on an AB Sciex Triple Quad 6500 mass spectrometer as previously described (Bachman et al., 2014). Results are expressed as percentage of total cytosine.

DNA and RNA extraction and BS-Seq Library Preparation
For each time point 3 biological replicates from 3 independent experiments were analysed. These consisted of MEFs, a day 6 FACS sorted refractory population (d6 Thy1+/SSEA1-: d6T+/S-) and cells prone to reprogramming (d6 Thy1-/SSEA1+: d6T-/S+), Dox-independent iPSCs at intermediate-late stages of reprogramming (d21 and d29 iPSCs) and established iPSCs (d60 iPSC), and a C57BL/6 background ESC line (TMBD10 - ESCs). Genomic DNA and total RNA was prepared using AllPrep DNA/ RNA mini kit (QIAGEN). For BS-Seq library preparation, DNA samples were fragmented by sonication (Covaris) and adaptor ligated using Illumina supplied methylated adaptors and NEBnext library preparation kit. Subsequently, DNA was bisulphite-treated using the TrueMethyl kit (Cambridge Epigenetix), according to the manufacturer's instructions. Final library amplification (11 cycles) was done using Kapa Uracil Plus (Kapa Biosytems), after which the libraries were bead-purified using 1x XP Ampure beads (Beckman Coulter). Libraries were sequenced on Illumina HiSeq.
For PBAT libraries, DNA samples were bisulphite converted using the EZ-DNA methylation direct kit. First strand synthesis was conducted as previously described (Smallwood et al., 2014), however, using F oligo 6N (CTACACGACGCTCTTCCGATCTNNNNNN) instead of a biotinylated 8N oligo. Samples were exonuclease treated. Second strand synthesis was conducted using a 8R oligo 6N (TGCTGAACCGCTCTTCCGATCTNNNNNN). Samples were amplified for 10 cycles using Kapa HotStart (Kapa Biosystems).

RNASeq Library Preparation
For each sample mRNA was purified from 50 ng of total RNA, using a Dynabeads® mRNA Purification Kit (Life Technologies) according to the manufacturer's instructions. This RNA was subsequently used for library preparation as described previously (Lin Wang et al., 2011). Libraries were amplified (14 cycles) and purified using 0.8x XP Ampure beads. Libraries were sequenced on Illumina HiSeq 2500.

BS-Seq Analysis
Raw sequence reads were trimmed to remove both poor quality calls and adapters using Trim Galore (Babraham Institute Bioinformatics). For PBAT libraries the 5' end of reads were also trimmed. Sequences were mapped to the mouse NCBIM37 genome using Bismark (Krueger and Andrews, 2011), and CpG methylation calls were extracted and analysed with SeqMonk and R. For PBAT analysis, 35k probes were designed across the whole genome and global methylation levels obtained. For BS-Seq, in order to obtain 3-fold coverage, replicates from

each time-point were pooled together. 50 CpG dinucleotide-containing probes were design across the whole genome and methylation over a given genomic feature was calculated by averaging the individual methylation levels of CpGs across these probes, only probes with reads in at least 10 CpGs were taken forward for analysis. CpG island (CGI) annotations were used based on pull down experiments (Illingworth et al., 2010). Promoters were defined as the region -1 kb to +500 bp of the transcription start site as annotated in NCBIM37, and were subdivided into CGI and non-CGI promoters based on whether they overlapped with a CGI. Repeat annotations were extracted from the UCSC RepeatMasker track (NCBIM37). ESC enhancer and super-enhancer (Whyte et al., 2013) and limb enhancer (Shen et al., 2012) coordinates were described previously. Background model was obtained by calculating the global mean for the ending methylation value for each starting methylation value. Afterwards, a binomial test was used on individual probes for their ending methylation measures against the mean for their starting methylation, to obtain p-value<0.05 DMRs. Differences in methylation at specific features were calculated and compared to a set of methylation matched random probes (MMRP). Probes from the reference sample whole genome with ±5% methylation were picked to create a subset of MMRP with an equal number of probes to the feature analysed. p-values in each feature were calculated using a pairwise t-test with a Benjamini-Hochberg correction. Targeted demethylation was considered only in features that deviated from the MMRP profile by at least 5% and had a significant p-value after Benjamini-Hochberg correction. Publically available datasets for oocyte, 2-cell and 4-cell embryos, ICM, Epiblast (Lu Wang et al., 2014), Epiblast E6.5, PGC E9.5, PGC E11.5 (Seisenberger et al., 2012), serum grown ESC, 2i grown ESCs (Ficz et al., 2013) were included in this analysis where indicated.

BS-Seq Amplicon assay

For BS-seq library preparation DNA samples were bisulphite-treated using the Epitect Kit (QIAGEN), according to the manufacturer's instructions. DNA was PCR-amplified using specific primers for 35 cycles. All PCR products for each sample were pooled together and bead-purified using 1x XP Ampure beads (Beckman Coulter). This pooled library was then amplified for additional 8 cycles using iPCRTag (Quail et al., 2012) primers. Libraries were sequenced on Illumina MiSeq. Primer design was carried out using MethPrimer (Li and Dahiya, 2002), and a 33bp adapter for high-throughput sequencing was added to the primer. Selected primers amplified specific pluripotency gene regulatory regions, highly significant DMRs at ESC enhancers or super-enhancers and limb enhancers.

RNASeq Analysis

RNA-Seq data was mapped to the mouse genome assembly (NCBIM37) using TopHat and gene models from Ensembl release 61. The number of reads mapping to each annotated mRNA transcript was quantified and counts were used in DESeq and Seqmonk to assess differentially expressed genes, during reprogramming. Expression clusters were defined by performing hierarchical clustering of transcripts based on a Pearson's correlation across all samples, and selecting groups which had a correlation coefficient greater than 0.7. Counts corrected for total read count with length correction (reads per kilobase per million, RPKM) were used to plot graphs and perform statistical tests using Prism 6 (GraphPad Software Inc., San Diego, CA, USA).

Gene expression analysis by qPCR

Total RNA was prepared using the Qiagen AllPrep DNA/RNA mini kit according to the manufacturers' instructions. cDNA was obtained by reverse transcription of 0.5 µg of RNA using SuperScript III reverse-transcriptase kit with random hexamer primers (Invitrogen), diluted 1:50 and used as a template in quantitative real-time PCR (qPCR) with a Bio-Rad C1000 Thermal Cycler. The relative abundance of each gene of interest was normalized to the average Ct of two housekeeping genes with consistent Ct values over all samples (Atp5b and Hsp90ab1) and calculated using the ddCt method.

Western blot (WB) analysis

For WB analysis, whole cell extracts were resolved by SDS-PAGE and blotted on PVDF membranes. Membranes were blocked in TBS-Tween (5% milk) and incubated overnight at 4°C with the indicated antibodies. After incubation with secondary antibodies, HRP conjugates were detected using an enhanced chemiluminescence kit (ECL Plus, Amersham Biosciences). The following primary antibodies were used for Western: Dnmt1 (ab92453, Abcam), UHRF1 (Santa Cruz Biotechnology, sc-98817). Secondary antibodies used: HRP-conjugated anti-mouse, anti-rabbit (Santa Cruz Biotechnology, sc-2005, sc-2004) and anti-rat (GE healthcare, NA935).

Immunofluorescence, microscopy and image analysis

Cells were fixed in 2% paraformaldehyde for 30 min. After fixation cells were permeabilised with PBS+0.5% Triton X-100 for 1 hour and blocked overnight. Cells were incubated with anti-Dnmt3b (ab13604, Abcam), Sox2 (ab97959, Abcam) for 1 hour, washed and incubated with secondary antibodies (Alexa Fluor). Nuclei were stained with DAPI. Single optical slices were captured with a Zeiss LSM 780 or phase contrast Nikon TS100.

Supplemental References

Bachman, M., Uribe-Lewis, S., Yang, X., Williams, M., Murrell, A., and Balasubramanian, S. (2014). 5-Hydroxymethylcytosine is a predominantly stable DNA modification. Nat Chem *6*, 1049–1055.

Illingworth, R.S., Gruenewald-Schneider, U., Webb, S., Kerr, A.R.W., James, K.D., Turner, D.J., Smith, C., Harrison, D.J., Andrews, R., and Bird, A.P. (2010). Orphan CpG Islands Identify Numerous Conserved Promoters in the Mammalian Genome. PLoS Genet *6*, e1001134.

Krueger, F., and Andrews, S.R. (2011). Bismark: a flexible aligner and methylation caller for Bisulfite-Seq applications. Bioinformatics *27*, 1571–1572.

Li, L.-C., and Dahiya, R. (2002). MethPrimer: designing primers for methylation PCRs. Bioinformatics *18*, 1427–1431.

Quail, M.A., Otto, T.D., Gu, Y., Harris, S.R., Skelly, T.F., McQuillan, J.A., Swerdlow, H.P., and Oyola, S.O. (2012). Optimal enzymes for amplifying sequencing libraries. Nat. Methods *9*, 10–11.

Smallwood, S.A., Lee, H.J., Angermueller, C., Krueger, F., Saadeh, H., Peat, J., Andrews, S.R., Stegle, O., Reik, W., Kelsey, G., 2014. Single-cell genome-wide bisulfite sequencing for assessing epigenetic heterogeneity. Nat. Methods 11, 817–820. doi:10.1038/nmeth.3035

Shen, Y., Yue, F., McCleary, D.F., Ye, Z., Edsall, L., Kuan, S., Wagner, U., Dixon, J., Lee, L., Lobanenkov, V.V., Ren, B., 2012. A map of the cis-regulatory sequences in the mouse genome. Nature 488, 116–120. doi:10.1038/nature11243

Whyte, W.A., Orlando, D.A., Hnisz, D., Abraham, B.J., Lin, C.Y., Kagey, M.H., Rahl, P.B., Lee, T.I., Young, R.A., 2013. Master Transcription Factors and Mediator Establish Super-Enhancers at Key Cell Identity Genes. Cell 153, 307–319.

doi:10.1016/j.cell.2013.03.035
Wang, Lin, Si, Y., Dedow, L.K., Shao, Y., Liu, P., Brutnell, T.P., 2011. A Low-Cost Library Construction Protocol and Data Analysis Pipeline for Illumina-Based Strand-Specific Multiplex RNA-Seq. PLoS ONE 6, e26426. doi:10.1371/journal.pone.0026426.s015
Wang, Lu, Zhang, J., Duan, J., Gao, X., Zhu, W., Lu, X., Yang, L., Zhang, J., Li, G., Ci, W., Li, W., Zhou, Q., Aluru, N., Tang, F., He, C., Huang, X., Liu, J., 2014. Programming and Inheritanceof Parental DNA Methylomes in Mammals. Cell 157, 979–991. doi:10.1016/j.cell.2014.04.017
Yeom, Y.I., Fuhrmann, G., Ovitt, C.E., Brehm, A., Ohbo, K., Gross, M., Hubner, K., Schöler, H.R., 1996. Germline regulatory element of Oct-4 specific for the totipotent cycle of embryonal cells. Development 122, 881–894.
Yoshimizu, T., Sugiyama, N., De Felice, M., Yeom, Y.I., Ohbo, K., Masuko, K., Obinata, M., Abe, K., Schöler, H.R., Matsui, Y., 1999. Germline-specific expression of the Oct-4/green fluorescent protein (GFP) transgene in mice. Dev. Growth Differ. 41, 675–684.
