# Supplementary material for: Gender Differences in Global but Not Targeted Demethylation in iPSC Reprogramming
Source: Cell Rep. 2017 Jan 31;18(5):1079–89. doi: 10.1016/j.celrep.2017.01.008 (PMC5300890; doi:10.1016/j.celrep.2017.01.008)
Supplement: Document S2. Article plus Supplemental Information [file mmc2.pdf]

Cell Reports

Report

Gender Differences in Global but Not Targeted Demethylation in iPSC Reprogramming

Graphical Abstract

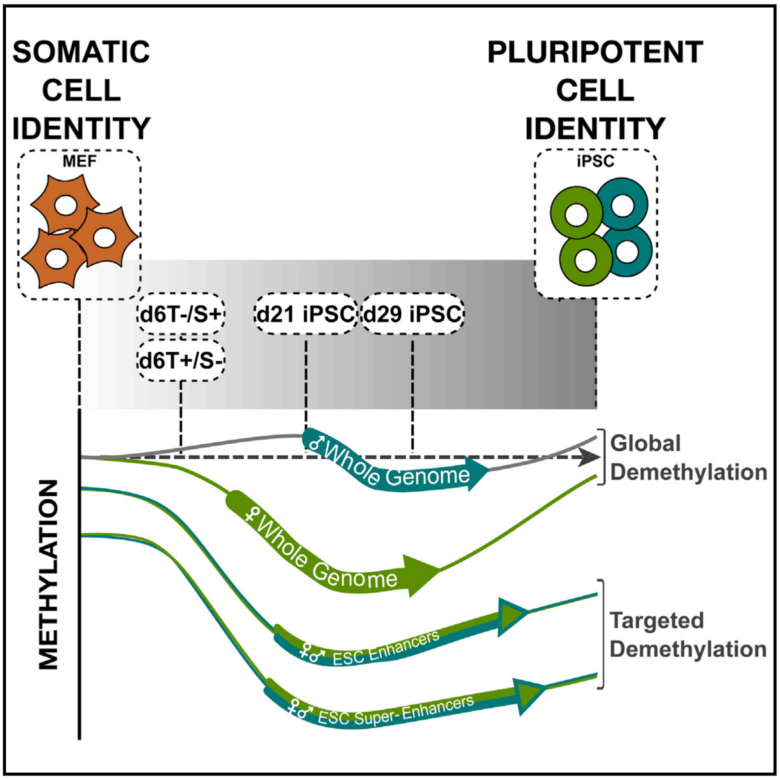

Highlights

- iPSC reprogramming induces transient genome-wide DNA demethylation
- Global demethylation is more pronounced in female iPSCs
- AID is a critical regulator of UHRF1 protein and genome-wide DNA demethylation
- Targeted DNA demethylation occurs at key regulatory regions and is uncoupled from global demethylation

Authors

Inês Milagre, Thomas M. Stubbs, Michelle R. King, ..., Simon R. Andrews, Wendy Dean, Wolf Reik

Correspondence

ines.milagre@babraham.ac.uk (I.M.), wolf.reik@babraham.ac.uk (W.R.)

In Brief

Milagre et al. find that two modes of DNA demethylation occur during primary iPSC reprogramming. Global DNA demethylation, more pronounced in female cells, is regulated by AID through UHRF1 and occurs transiently at intermediate-late stages of reprogramming. Targeted DNA demethylation, by contrast, is important in establishing hypomethylation at enhancers of pluripotency genes and occurs similarly in female and male cells.

Accession Numbers

GSE69823

Milagre et al., 2017, Cell Reports *18*, 1079–1089
January 31, 2017 © 2017 The Author(s).
http://dx.doi.org/10.1016/j.celrep.2017.01.008

CellPress

OPEN ACCESS
CellPress

Gender Differences in Global but Not Targeted Demethylation in iPSC Reprogramming

Inês Milagre,[1,7,*] Thomas M. Stubbs,[1] Michelle R. King,[1] Julia Spindel,[1] Fátima Santos,[1] Felix Krueger,[2] Martin Bachman,[3,4] Anne Segonds-Pichon,[2] Shankar Balasubramanian,[3,4] Simon R. Andrews,[2] Wendy Dean,[1] and Wolf Reik[1,5,6,8,*]

[1]Epigenetics Programme
[2]Bioinformatics Group
The Babraham Institute, Cambridge CB22 3AT, UK
[3]Department of Chemistry, University of Cambridge, Cambridge CB2 1EW, UK
[4]Cancer Research UK Cambridge Institute, University of Cambridge, Cambridge CB2 0RE, UK
[5]Centre for Trophoblast Research, University of Cambridge, Cambridge CB2 3EG, UK
[6]Wellcome Trust Sanger Institute, Hinxton CB10 1SA, UK
[7]Present address: Instituto Gulbenkian de Ciência, Rua da Quinta Grande 6, 2780-156 Oeiras, Portugal
[8]Lead Contact
*Correspondence: ines.milagre@babraham.ac.uk (I.M.), wolf.reik@babraham.ac.uk (W.R.)
http://dx.doi.org/10.1016/j.celrep.2017.01.008

SUMMARY

Global DNA demethylation is an integral part of reprogramming processes in vivo and in vitro, but whether it occurs in the derivation of induced pluripotent stem cells (iPSCs) is not known. Here, we show that iPSC reprogramming involves both global and targeted demethylation, which are separable mechanistically and by their biological outcomes. Cells at intermediate-late stages of reprogramming undergo transient genome-wide demethylation, which is more pronounced in female cells. Global demethylation requires activation-induced cytidine deaminase (AID)-mediated downregulation of UHRF1 protein, and abolishing demethylation leaves thousands of hypermethylated regions in the iPSC genome. Independently of AID and global demethylation, regulatory regions, particularly ESC enhancers and super-enhancers, are specifically targeted for hypomethylation in association with transcription of the pluripotency network. Our results show that global and targeted DNA demethylation are conserved and distinct reprogramming processes, presumably because of their respective roles in epigenetic memory erasure and in the establishment of cell identity.

INTRODUCTION

Induced pluripotent stem cell (iPSC) technology holds unparalleled promise for research, tissue engineering, and regenerative medicine. Reprogramming is a continuous process characterized by the stepwise activation of fundamental pluripotency genes (Brambrink et al., 2008; Stadtfeld et al., 2008) and the silencing of somatic cell-of-origin genes (Buganim et al., 2012). This complex remodeling of transcriptional networks is associated with reprogramming of the epigenome (Brambrink et al., 2008; Sridharan et al., 2009; Stadtfeld et al., 2008), which is the ensemble of DNA or chromatin modifications linked with gene expression states without affecting DNA sequence. Specific histone marks are lost (Chen et al., 2013b; Golipour et al., 2012; Yang et al., 2014) or acquired (Buganim et al., 2012; Cacchiarelli et al., 2015; Mikkelsen et al., 2008) during reprogramming, and activation of microRNAs (Polo et al., 2012) and long noncoding RNAs (Kim et al., 2015) at defined stages is also important. A critical role for DNA demethylation in complete and robust reprogramming of terminally differentiated cells has been proposed (Papp and Plath, 2013); however, the molecular mechanisms underlying this epigenetic process and its dynamics at different stages during reprogramming are poorly understood. It is also not clear to what extent demethylation is involved in the creation of a pluripotent cell identity and whether it may also be needed to remove epigenetic memory.

iPSCs have been shown to tolerate global hypomethylation, just as embryonic stem cells (ESCs) do (Wernig et al., 2007), and the efficiency of obtaining these cells is improved by treatment with 5-azacytidine (Mikkelsen et al., 2008). Tet family dioxygenases hydroxylate 5-methylcytosine (5mC) and enhance reprogramming efficiency (Costa et al., 2013; Doege et al., 2012; Hu et al., 2014). The cytosine deaminase activation-induced cytidine deaminase (AID) stabilizes the pluripotent phenotype (Kumar et al., 2013) and is needed for demethylation of specific promoters in heterokaryon reprogramming (Bhutani et al., 2010), but the extent, timing, and mechanisms of this demethylation in iPSC reprogramming are not known.

Despite demethylation being critical, there are only a few comprehensive studies of DNA methylation dynamics during the reprogramming process and none in a primary reprogramming system. Polo et al. (2012), using a methylation array to study promoter regions, showed that demethylation occurs gradually, while new methylation marks are gained only late in reprogramming. More recently, Lee et al. (2014a) performed

This is an open access article under the CC BY license (http://creativecommons.org/licenses/by/4.0/).

genome-wide analyses in intermediates of F-class cells (an alternative pluripotent state, dependent on continuous high expression levels of the Yamanaka Factors [Oct3/4, Sox2, Klf4, cMyc] OSKM) and one late time point where OSKM were no longer exogenously expressed. They demonstrated that hypomethylated differentially methylated regions (DMRs) are highly enriched in H3K4me3 and H3K27me3 and that the majority of these overlap with specific transcription factor binding sites. However, it is not clear how DNA methylation marks at regulatory regions are remodeled during primary iPSC reprogramming and how this is linked to the establishment of the pluripotent gene expression program. Here, we perform a comprehensive genome-wide in-depth analysis of the dynamics of DNA demethylation and its link to transcription during primary mouse embryonic fibroblast (MEF) reprogramming to iPSCs. We demonstrate that both female and male cells undergo global hypomethylation of the genome, which is likely to be important for the removal of epigenetic memory. Independently, targeted loss of DNA methylation marks at critical regulatory regions is necessary for the establishment of cell identity. We show that global demethylation is more pronounced in female cells, while targeted demethylation at regulatory regions is evident in both female and male cells. Finally, we find that AID plays a key role in global demethylation and epigenetic memory erasure. Surprisingly, this occurs at the level of regulation of UHRF1 protein, an essential component of the DNA methylation maintenance machinery, recently also shown to be regulated during global demethylation in ESCs transitioning from serum to 2i (von Meyenn et al., 2016).

RESULTS

iPSC Reprogramming Triggers Transient Global DNA Hypomethylation

To enable dynamic methylation profiling, reprogramming intermediates were analyzed at defined time points (Figure 1A; for a detailed description, see Experimental Procedures). Oct4-GFP MEFs were reprogrammed in low oxygen (5% O_2), known to improve reprogramming (Yoshida et al., 2009), in serum medium and using an inducible piggybac system. This allowed us to obtain and pick colonies as early as day 6 (d6) after induction of OSKM by doxycycline (Dox) and analyzing these same clones over time, allowing for the characterization of intermediate time points that are not accessible through primary reprogramming carried out in normoxic conditions (Figure 1A). Female and male iPSC clones at intermediate-late stages of reprogramming (d21 and d29 iPSCs) already express the majority of the pluripotency factors, but in contrast to established iPSCs (d60 iPSCs) female cells are still in the process of downregulating Xist and thus in the process of completing X chromosome reactivation (Figure S1A). Dox-independent GFP-positive colonies at d21 that showed expression of key pluripotency markers and the ability to differentiate into the three germ layers as well as a normal karyotype by d60 (Figures S1B–S1D) were used in subsequent analyses. Embryonic stem cells (ESCs) were included in the analysis as a control for pluripotent cells.

Notably, liquid chromatography mass spectrometry (LC-MS) revealed substantial global demethylation in intermediate-late stages of reprogramming in female cells (Figure 1B). While methylation levels comparable to MEFs (3.0%) were maintained at d6 (d6T^+/S^-: 3.0% and d6T^-/S^+: 3.1%), in intermediate-late stages there was a significant decrease in 5-methylcytosine (5mC) levels (d21 iPSCs: 2.7% and d29 iPSC: 2.4%, $p < 0.05$: MEFs versus d29 iPSCs, ANOVA with Dunnett's correction). However, this global hypomethylation was transient with established iPSCs's 5mC levels (3.8%) similar to those of primed ESCs (3.9%). Global demethylation during reprogramming of female cells was confirmed by whole-genome bisulphite sequencing (WGBS), which revealed a substantial drop in CpG methylation levels from 68% in MEFs to 45% in d29 iPSCs, with subsequent remethylation to 67% in d60 iPSCs (Figures 1C, S1E, and S1G). Interestingly, during reprogramming of male cells this global demethylation was not as marked as in female cells (Figures 1D, S1F, and S1G).

The methylation dynamics during iPSC reprogramming closely resembled the transient loss of DNA methylation marks in early embryo development, where DNA methylation is globally lost from the oocyte (52%) to the 2- and 4-cell stage (47% and 38%) until it reaches very low levels (20%) in the inner cell mass (ICM) at the blastocyst stage. Methylation marks are then regained by the E6.5 epiblast stage (61%) (Figures 1E and S1H). Moreover, global demethylation was uncoupled from transcriptional regulation (Figure S1I) as previously reported in primordial germ cell (PGCs) development, and in the transition from primed to naive ESCs (Seisenberger et al., 2012; Ficz et al., 2013).

These results show that global DNA demethylation occurs in the intermediate-late stages of reprogramming and that female and male cells undergo different modulation of DNA methylation dynamics during reprogramming.

Stable Targeted DNA Demethylation Occurs at Pluripotency Regulatory Regions and Correlates with Expression of the Pluripotency Network

In order to integrate and validate the differences in global methylation levels observed between the discrete time points within our experiment, the development of specific analytical approaches was required. These approaches also allowed insights into other reprogramming systems, thus highlighting their usefulness (for detailed description, see Supplemental Experimental Procedures). The first approach employs a background model to correct for global methylation differences, which allowed us to confidently call differentially methylated regions (DMRs) from MEFs to established iPSCs. Gene bodies, intergenic regions, and long interspersed nuclear elements (LINEs) and short interspersed nuclear elements (SINEs) have a similar methylation profile to the genome as a whole and hence follow the global demethylation and remethylation event (Figures 2A and S2A). In contrast, intracisternal A particle (IAP) retrotransposons and limb enhancers (as an example of a tissue-specific enhancer) are protected from demethylation, with many DMRs being hypermethylated in the established iPSCs. Notably, pluripotency regulatory regions such as promoters, ESC enhancers, and super-enhancers (SEs) are specifically targeted for demethylation, with the majority of these DMRs being hypomethylated over and above the genome average (Figures 2A and S2A). The importance of hypomethylation for enhancer and SE function in

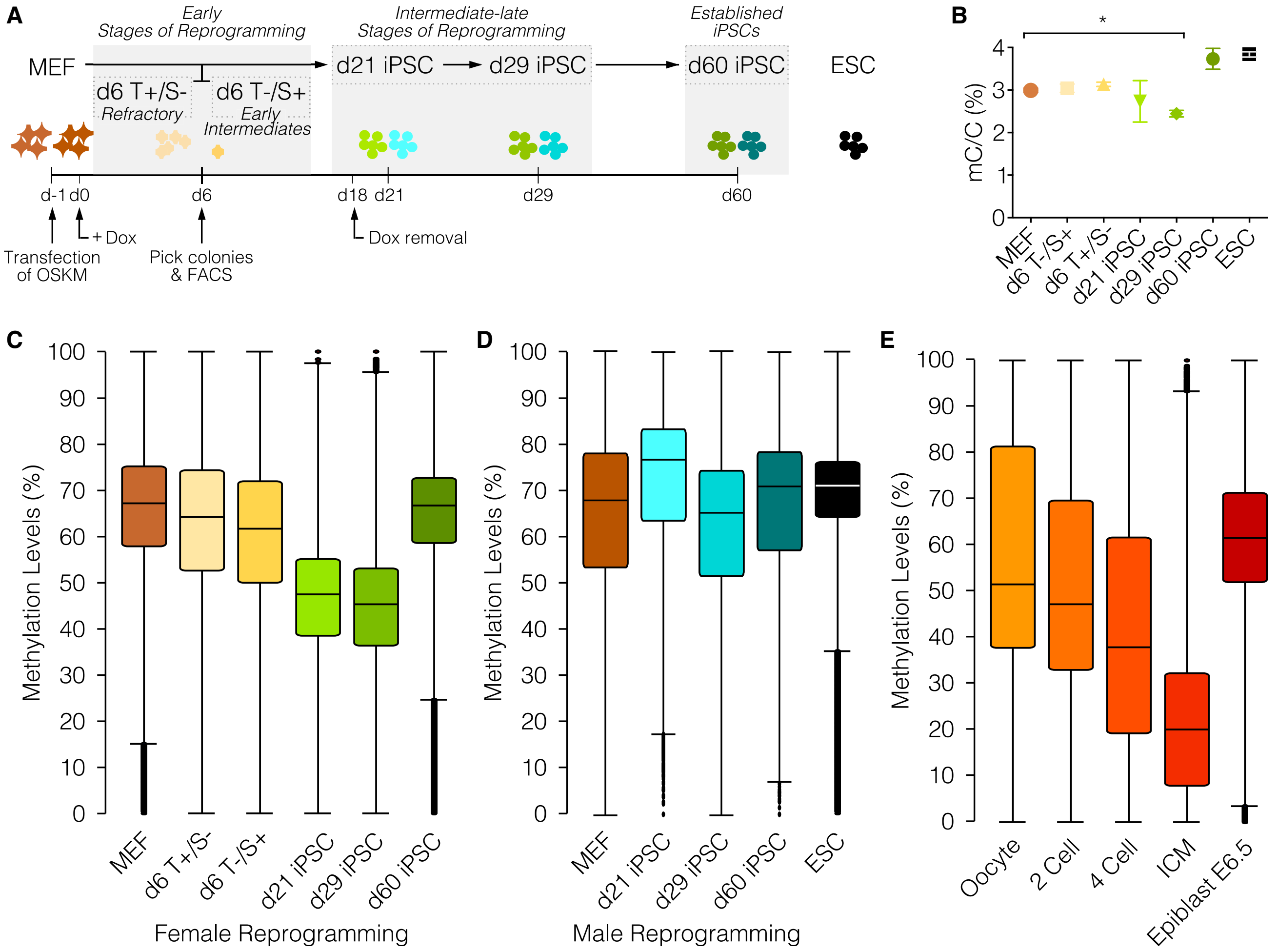

Figure 1. Global DNA Demethylation Dynamics during iPSC Reprogramming

(A) Scheme of the reprogramming system. d6T^{+}/S^{-} and d6T^{-}/S^{+}: Thy1 (T) and SSEA1 (S) FACS cells at day 6 of reprogramming. For detailed description, refer to Experimental Procedures.

(B) Global 5mC levels measured by LC-MS. Results are expressed as percentage of total cytosines and data are represented as mean ± SEM p values shown are the result of an ANOVA with Dunnett's correction.

(C–E) CpG methylation levels, as assessed by BS-seq, during reprogramming of (C) female, (D) male cells, and (E) pre-implantation embryo (oocyte, 2- and 4-cell embryos, ICM, and epiblast). (C–E) Plot displays the median (bar), inter-quartile range (box), and maximum and minimum (whiskers).

See also Figure S1.

ESCs is well documented (Ding et al., 2015; Stadler et al., 2011; Wiench et al., 2011) but has not been described in iPSC reprogramming. To validate the DMRs found from WGBS, an amplicon-based assay was designed. This assay allowed the methylation dynamics of selected regions to be interrogated at >1,000-fold sequencing depth. The results closely matched those obtained from our low sequencing depth (3-fold) WGBS data, showing that such coverage nevertheless provided robust methylome information on individual loci (Figure S2B). Unlike global demethylation, targeted demethylation occurs at the same regions and to a similar extent during female and male somatic cell reprogramming (Figure 2A).

Hierarchical clustering of RNA sequencing (RNA-seq) data of female cells during reprogramming showed that differentially expressed genes from MEFs to established iPSCs fell into five distinct expression clusters (Figure S2C) similar to those previously described (O'Malley et al., 2013). Notably, genes in cluster II (upregulated during reprogramming—including the pluripotency network genes) are enriched for hypomethylated DMRs in non-CGI promoters, ESC enhancers, and SEs (Figure 2B). Conversely, limb enhancer DMRs, which remain hypermethylated, are absent from this cluster. In contrast, genes in cluster V (downregulated during reprogramming) are exclusively enriched for DMRs at limb enhancers (Figure 2B). Similar results were seen for d6T^{-}/S^{+} and ESCs (Figure S2D), showing that upregulation of pluripotency genes precedes global demethylation and is influenced by targeted demethylation at ESC enhancers and super-enhancers. Instructive examples of changes in regulatory regions of individual genes are shown in Figures 2C, 2D, S2E, and S2F. These results show that demethylation at specific

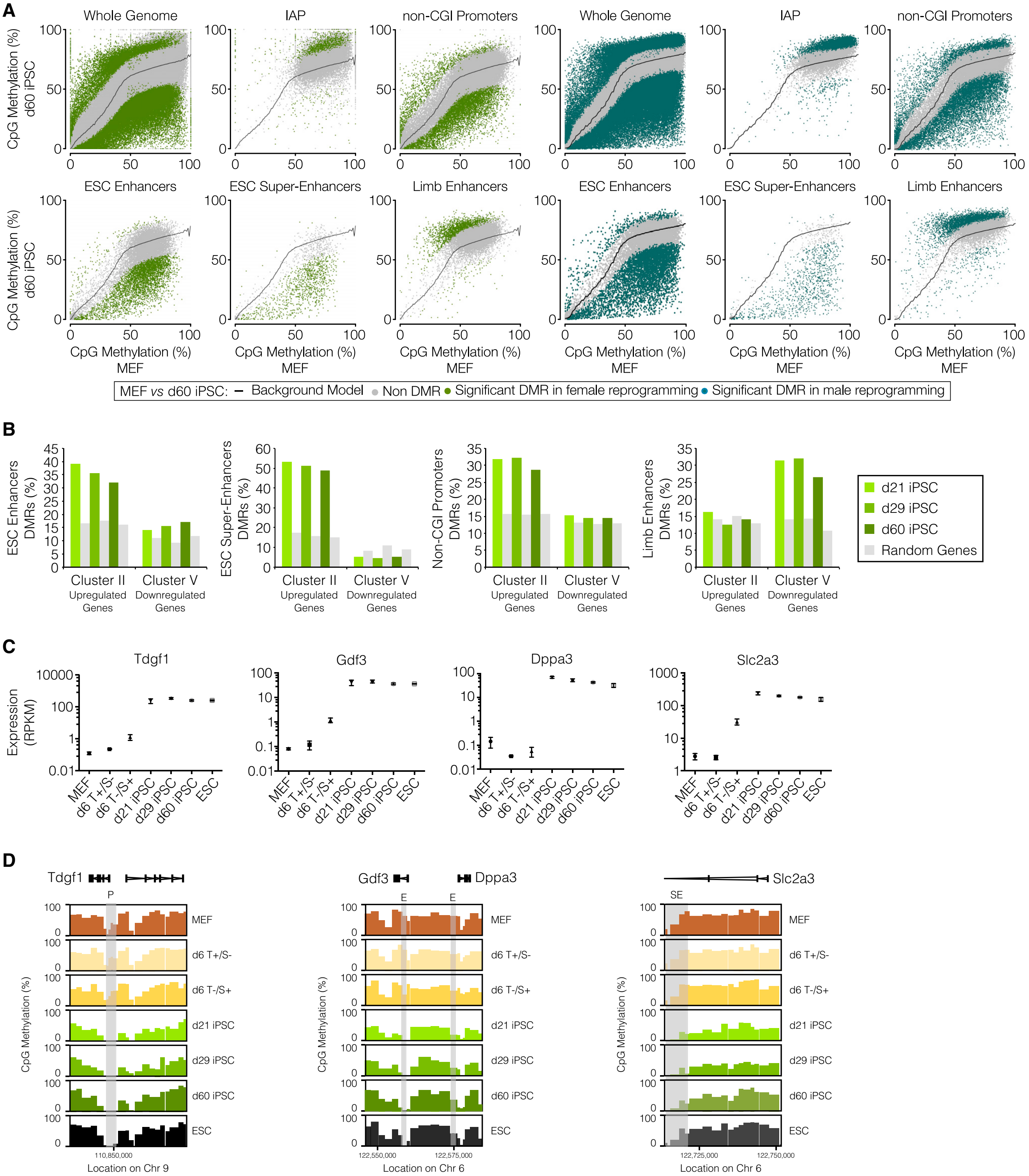

Figure 2. Correlation of DNA Methylation at Different Regulatory Regions and Gene Expression

(A) Scatterplot of DNA methylation levels of individual probes genome-wide, showing whole genome and different genomic features of MEFs and d60 iPSCs. Dots represent individual 50 CpG probes—significant differentially methylated regions (DMRs) are represented in green (female cell reprogramming) or blue (male cell reprogramming). Background model depicted as a black line.

(B) Percentage of DMRs at regulatory regions that overlap with specific gene clusters, compared to random sets with the same number of genes (in gray).

(legend continued on next page)

regulatory regions is important for the upregulation of the pluripotency network genes. In established female and male iPSCs, depletion of methylation in ESC enhancers and non-CGI promoters was more pronounced in highly and very highly expressed genes (Figure 3A), in agreement with previous studies in ESCs (Lister et al., 2009; Stadler et al., 2011).

We developed a second analytical approach that utilizes methylation-matched random probes (MMRPs) to account for global methylation differences, which allows for clear visualization of methylation differences during iPSC reprogramming (relative to MEFs). This approach validates the targeted demethylation findings in both female and male cells (Figures S3A and S3B), and more importantly it allows for statistical significance to be calculated after grouping of the differences elicited by reprogramming, irrespective of the differences in the process or the genome coverage and sequencing depth of the data. Based on these analyses, we observed that DNA demethylation at ESC enhancers and super-enhancers is already evident in the intermediate-late stages of reprogramming but becomes yet more pronounced in d60 iPSCs (Figures 3B, 3C, S3C, and S3D). Notably, we identified targeted demethylation at these same regulatory regions in pre-implantation embryos and during the serum to 2i transition (Figure 3D) while it was absent in unipotent PGCs (Surani, 2012). These results reveal a conserved targeted demethylation signature during reprogramming to pluripotent cell identity in vitro and in vivo, which seems to be independent of the extent of global DNA demethylation.

AID Regulates UHRF1 and Global Demethylation during iPSC Reprogramming

To understand the molecular regulation of global demethylation associated with female cell reprograming, we assessed the expression levels of genes implicated in DNA methylation and demethylation. As previously shown (Buganim et al., 2012; Polo et al., 2012), all *Dnmt* genes were significantly upregulated upon reprogramming (Figure S4A). Importantly, we also confirmed upregulation of DNMT1 and DNMT3b proteins (Figures 4A and S4B). However, we observed one notable exception to this pattern UHRF1, responsible for the recruitment of DNMT1 to hemi-methylated DNA (Bostick et al., 2007; Sharif et al., 2007). While its transcription was upregulated by reprogramming, we observed substantially reduced protein levels at the stages associated with global hypomethylation (Figures 4A and 4B), when compared to fully reprogrammed iPSCs.

AID has been previously implicated in iPSC reprogramming, but the timing, mechanisms, and extent of demethylation it may regulate are unknown (Bhutani et al., 2013; Kumar et al., 2013). It was therefore interesting to note that *Aid* expression peaked precisely in d29 iPSCs (Figure 4C) when DNA methylation levels are lowest. Moreover, global demethylation during reprogramming of female Aid knock-out (AidKO) MEFs was much less substantial and was delayed when compared to wild-type (WT) female cell reprogramming (Figures 4D, 4E, S4C, and S4D). Typical reprograming-induced demethylation in female cells was partially rescued by re-expression of either the wild-type or a catalytically mutant isoform of AID, but not by expression of an empty vector (Figure 4F). These results show the importance of AID in regulating global DNA demethylation during reprogramming and that this regulation is independent of the deaminase activity of AID.

Given the marked abrogation of demethylation observed during reprograming of female AidKO MEFs, and the downregulation of UHRF1 protein associated with reprograming and global hypomethylation in WT cells, we compared UHRF1 protein levels during WT versus AidKO cell reprograming. Intriguingly, deficiency in AID prevented the downregulation of UHRF1 protein (Figures 4G and 4H). In addition, overexpression of AID (both WT and deaminase mutant) in AidKO reprogramming cells led to a significant decrease in UHRF1 protein levels (Figures 4I and 4J), consistent with a role for AID in regulating its abundance at a posttranscriptional level and reinforcing its importance in regulating global demethylation. We note in this respect the differential expression of genes involved in ubiquitination (which is known to regulate UHRF1 [Chen et al., 2013a]) between WT and AidKO d29 iPSCs (Figure S4E), which included *Lonrf3*, *Mdm2*, *Usp48*, *Pramel7*, *Rnf32*, *Shprh*, and *Trim17* among others.

It is notable that despite the profound defect in the transient global demethylation associated with reprograming, we detected no differences in global methylation levels between WT and AidKO d60 iPSCs, presumably due to the de novo methylation wave that takes place at the later stages of reprograming. However, targeted demethylation at ESC-specific enhancers and super-enhancers was not affected by lack of AID (Figure 4K) consistent with the fact that in general activation of the pluripotency transcriptional program occurred normally in AidKO iPSCs (Figure S4F). This is consistent with the mild effects of AID deficiency on obtaining iPSCs (Habib et al., 2014; Shimamoto et al., 2014). However we did identify more than 17,000 DMRs in AidKO iPSCs, most of which (72%) were hypermethylated (Figure S4G). These hypermethylated DMRs occur throughout the genome and in all genomic features, consistent with a global effect of AID. Additionally, these cells appear to have impaired differentiation potential, as they are unable to upregulate several differentiation markers at the same levels as WT cells (Figure S4H). These findings reconcile previous observations on AidKO iPSCs (Kumar et al., 2013), showing that global demethylation is mechanistically uncoupled from targeted demethylation and is necessary for the erasure of epigenetic memory.

Our data also showed significant upregulation of the ten-eleven Translocation (Tet) *Tet1* and *Tet2,* and *Tdg* genes in d29 iPSCs (Figure S4I) that continue to be highly expressed in fully reprogrammed iPSCs. Hydroxymethylation levels were low in MEFs and d6T$^+$/S$^-$ cells, in contrast to d6T$^-$/S$^+$ cells that have hydroxymethylation levels similar to primed ESCs

(C) Expression profiles (reads per kilobase pre million mapped reads [RPKM]) for *Tdgf1*, *Gdf3*, *Dppa3*, and *Slc2a3*.
(D) Example of BS-seq profile for *Tdgf1*, *Gdf3*, *Dppa3*, and *Slc2a3* at promoter, enhancer, and SE regions. Methylation levels of individual probes, between 0% and 100% are shown. Shadowed areas highlight promoter (P), enhancer (E), or super-enhancer (SE) regions.
See also Figure S2.

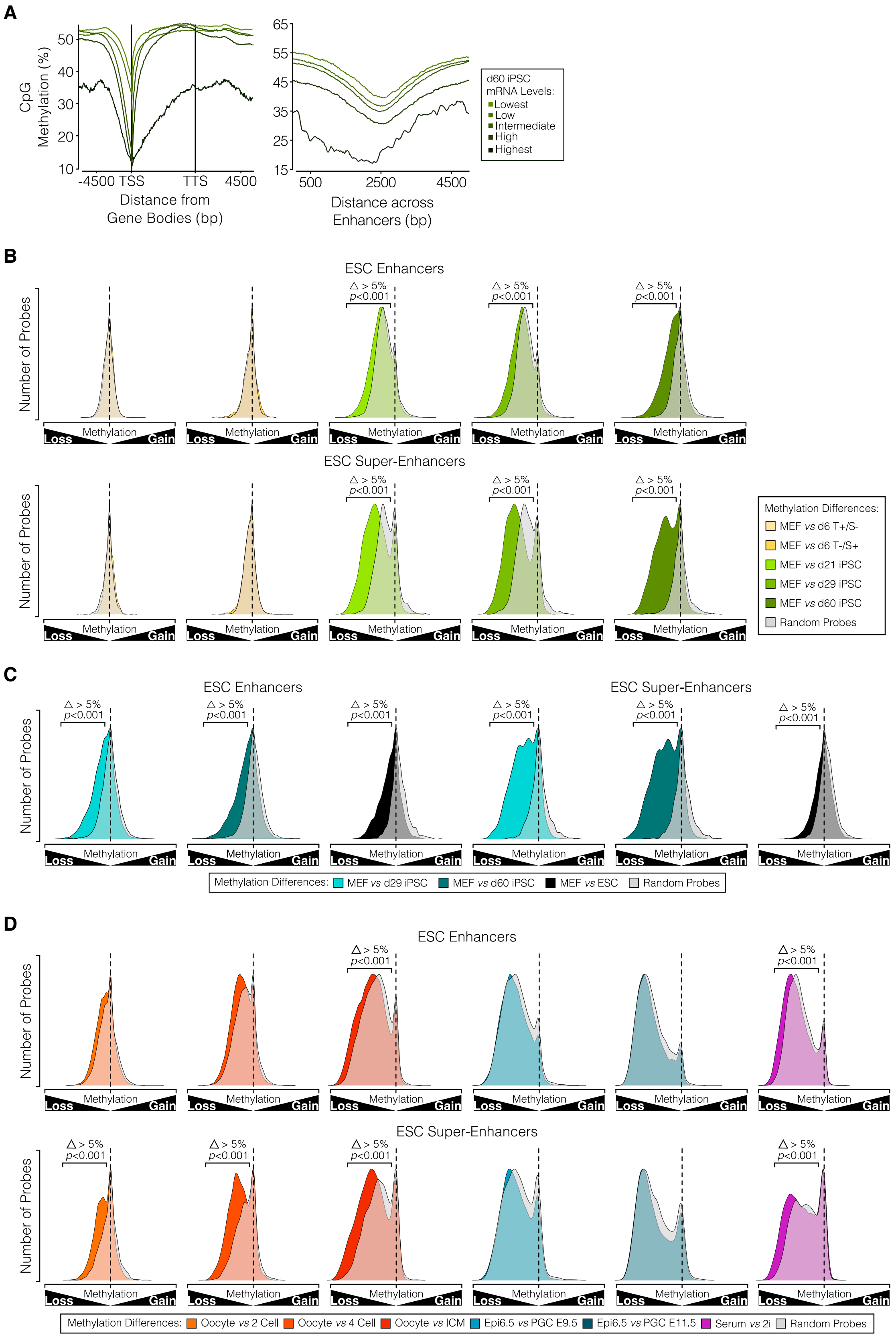

(legend on next page)

(Figure S4J). This increase in 5-hydroxymethylcytosine (5hmC) correlates with the observed increase in expression of the *Tet* enzymes, consistent with their role in controlling MET (Hu et al., 2014). Furthermore, the expression dynamics of *Tet1*, *2* and *Tdg* are consistent with a possible role in targeted but not in global demethylation.

Finally, based on the observation that female and male cells undergo global DNA demethylation to different extents, and when female cells are devoid of AID they show an extent of global demethylation similar to male WT cells, we investigated the status of X chromosome reactivation. We observed that AidKO female cells are able to reactivate the X chromosome, just like WT cells (Figure S4K). Furthermore, to exclude that this could be a reprogramming system-specific effect, we reprogrammed fibroblasts from one male and four female human donors. Human cells also undergo global demethylation during reprogramming (Figure S4L). However, this demethylation is not as profound as in female mouse cells, resembling more closely the male mouse global DNA methylation profile. Since human cells do not robustly reactivate the X chromosome during iPSC reprogramming (Tchieu et al., 2010), we investigated whether this was the case in our reprogramming system. We observed that our human cells were not able to reactivate the X chromosome and *Xist* expression was maintained (Figures S4M and S4N). Moreover, we observed that *Aid* is expressed at d11, when methylation levels are lower (Figure S4O).

These results suggest that global DNA demethylation during reprogramming is mainly achieved by passive demethylation, similar to what has been reported in other reprogramming processes (Seisenberger et al., 2012). Furthermore, AID can influence the global methylation levels during reprograming by regulating the protein levels of UHRF1, and thus the efficiency of recruitment of the maintenance methylation machinery. Additionally, the extent of global DNA demethylation is not dependent on the reprogramming system or species but seems to be influenced by the capacity of cells to reactivate the X chromosome (Figure 4L).

DISCUSSION

The extent and role of DNA methylation remodeling during the reprogramming of somatic cells to pluripotency are poorly understood. Our detailed and comprehensive study reveals that iPSCs undergo transient global demethylation during reprogramming and that stable targeted demethylation occurs in parallel to the global one. Notably, we show that the targeted and global demethylation processes are mechanistically uncoupled and that upregulation of pluripotency genes precedes and is not dependent on the extent of global demethylation. Targeted demethylation establishes a unique epigenetic pluripotency signature, which is broadly conserved in other reprogramming processes. An important caveat is that there are gender-specific differences in the extent to which the genome demethylates globally. In female cells, where DNA demethylation is more pronounced, downregulation of UHRF1 protein, through an AID-dependent mechanism, facilitates global but not targeted demethylation (Figure 4L). Moreover, our results clearly show that cells lacking AID-mediated global demethylation have an impaired differentiation potential, showing that AID is important for epigenetic memory erasure but not for the establishment of pluripotent cell identity.

Global DNA demethylation occurs in early embryos, during PGC development and in naive ESCs in both mouse and human (von Meyenn and Reik, 2015) and has consequently been proposed to be a conserved and obligate feature of reprogramming (Lee et al., 2014b; Nashun et al., 2015). Here, we show that mouse female and male cells undergo different levels of genome-wide demethylation during iPSC reprogramming. Our results, extrapolated from human cell reprogramming, point to a role for X chromosome reactivation in influencing these differences. This is consistent with mouse female ESCs having lower global methylation levels than male ones (Zvetkova et al., 2005) and with a recent report in PGC-like cell induction, where female cells undergo DNA methylation reprogramming similar to male cells, however, with more pronounced global changes (Shirane et al., 2016).

We reprogram MEFs to iPSCs in the presence of serum, which in ESCs results in high global methylation levels similar to those of somatic cells (Ficz et al., 2013; Habibi et al., 2013; Leitch et al., 2013). Hence despite high levels of de novo methyltransferases, controlled downregulation of UHRF1 protein seems critical for global demethylation. We have recently shown that UHRF1 is also regulated at the protein level when mouse ESCs are transitioned from serum to 2i (von Meyenn et al., 2016). This potentially provides a unifying theme for genome-wide demethylation mechanisms, which in mice and humans are characterized by disabling of the UHRF1/DNMT1 system, including by posttranscriptional regulation of *Uhrf1* (Seisenberger et al., 2012; Sugawa et al., 2015).

The role of AID in DNA demethylation and reprogramming in vivo and in vitro has been puzzling with the majority of studies demonstrating that it plays a role in demethylation (Bhutani et al., 2010, 2013; Kumar et al., 2013; Popp et al., 2010; Santos et al., 2013) but that it has mild impact on iPSC reprogramming (Habib et al., 2014; Shimamoto et al., 2014). Our results clearly show that AID plays a major role in global DNA demethylation, and unexpectedly this seems to be brought about by its negative regulation of UHRF1 protein levels, suggesting a novel role for AID in

Figure 3. DNA Demethylation Dynamics at Specific Genomic Features and in Different Reprogramming Processes

(A) CpG methylation levels for promoter and enhancer regions of genes showing different expression levels in female d60 iPSCs.

(B and C) Density plots of methylation differences from MEFs to each time point in reprogramming of (B) female and (C) male cells and ESCs, at specific features shown by opaque plots, overlaid by MMRP transparent gray density plot.

(D) Density plots of methylation differences at specific features, from oocyte to each time point in pre-implantation embryo, epiblast to PGCs and serum to 2i ESCs, shown by opaque plots; overlaid by MMRP transparent gray density plot. (B–D) Analyses were performed for ESC enhancers and SE Δ denotes a minimum 5% difference between data and MMRP profile. p values shown are the result of a pairwise t test with a Benjamini-Hochberg correction.

See also Figure S3.

OPEN ACCESS
CellPress

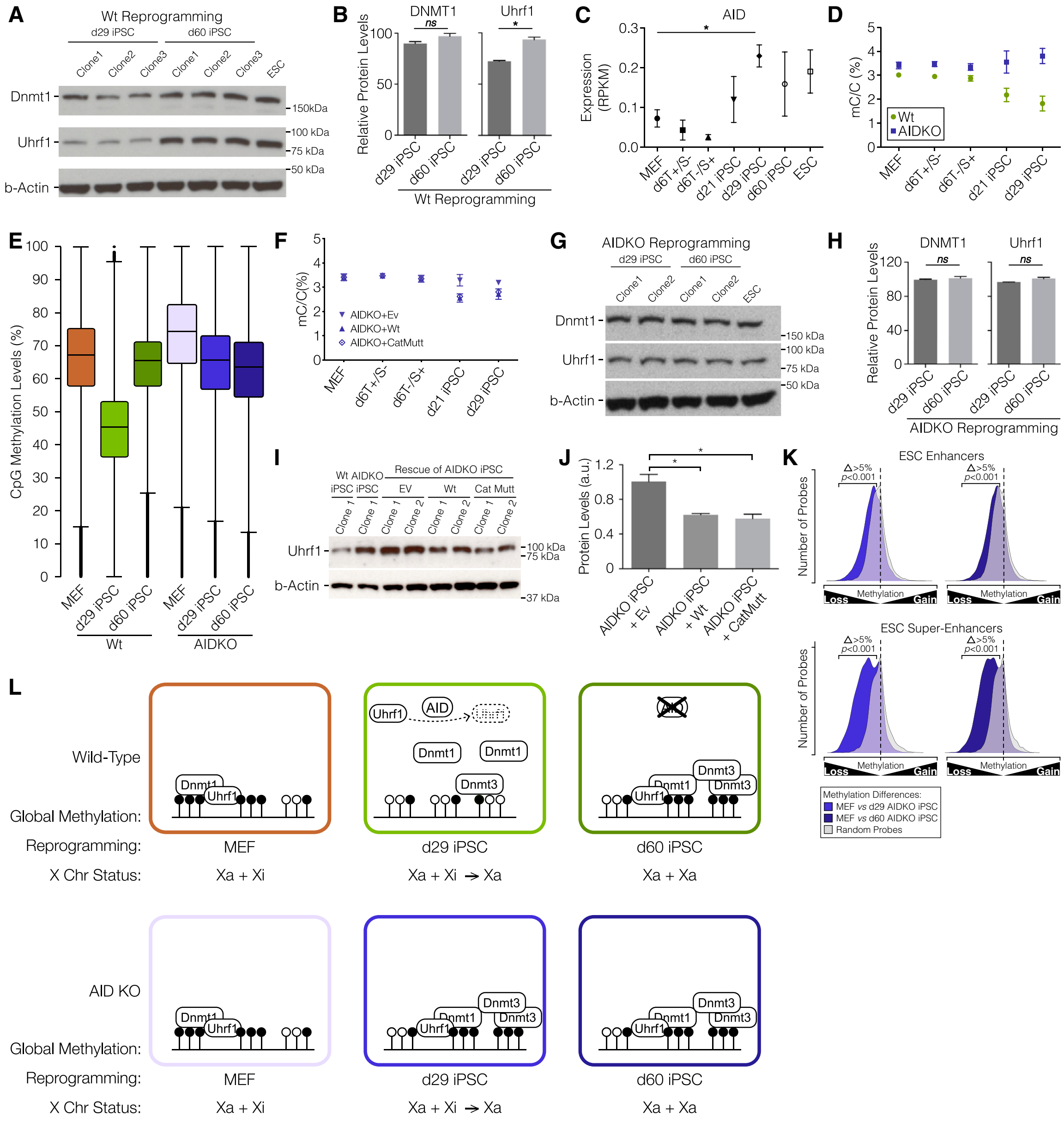

Figure 4. Mechanisms of DNA Demethylation

(A) WB showing levels of DNMT1 and UHRF1 in WT iPSCs at d29 and d60. β-actin was used as a loading control.

(B) Quantitation of DNMT1 and UHRF1 WB bands, relative to ESCs levels.

(C) Expression profile (RPKM) for *Aid*. p values shown are the result of two-tailed t tests from MEFs to d29 iPSCs.

(D) Global 5mC levels, measured by LC-MS. Results are expressed as percentage of total cytosine. Data are represented as mean ± SEM. Shown are results for reprogramming of WT and AidKO MEFs.

(E) Global CpG methylation levels, as assessed by BS-seq, for every time point during AidKO MEF reprogramming. Plot displays the median (bar), inter-quartile range (box), and maximum and minimum (whiskers).

(F) Global 5mC levels, measured by LC-MS. Results are expressed as percentage of total cytosine. Data are represented as mean ± SEM. Shown are results for AidKO MEFs reprogrammed with the OSKM plus an empty vector (EV), a vector containing AID WT cDNA (AIDWt), or a vector containing AID catalytic mutant cDNA (AIDCatMutt).

(G) WB showing levels of DNMT1 and UHRF1 in AidKO iPSCs at d29 and d60. β-actin was used as a loading control.

(H) Quantitation of DNMT1 and UHRF1 WB bands, relative to ESCs levels.

(I) WB showing levels of UHRF1 in WT and AidKO iPSCs d29 and clones rescued with EV, AIDWtor AIDCatMutt. β-actin was used as a loading control.

(J) Quantitation of UHRF1 WB bands (arbitrary units [a.u.]).

(legend continued on next page)

posttranslational regulation of UHRF1. Known mechanisms of UHRF1 regulation that can affect DNA methylation include ubiquitination among others (Tauber and Fischle, 2015). We note in this respect that several ubiquitination and deubiquitination enzymes are differentially expressed in iPSCs with and without AID, and that AID itself interacts with a ubiquitin ligase (Sun et al., 2013).

Targeted demethylation (over and above the global demethylation) occurs at ESC-specific enhancers and super-enhancers to a similar extent in female and male cells, and this is conserved in other reprogramming processes in which pluripotent cell identity is achieved. These regions share the characteristics of being CpG-poor and transcription factor (TF) binding-rich regions, characteristics that have been proposed to play a role in focal or targeted demethylation (Soufi et al., 2012; Stadler et al., 2011). TET proteins have also been implicated in reprogramming (Nashun et al., 2015), as well as in targeted enhancer (Pastor et al., 2013) and super-enhancer (Ding et al., 2015) demethylation in ESCs. However, Tet enzymes are needed specifically for activation of microRNAs essential for iPSC derivation, and it is possible to obtain fully reprogrammed iPSCs from *Tet1-3* triple-knockout MEFs after ectopic expression of miR200c (Hu et al., 2014). Nevertheless, the dynamics of *Tet* enzyme expression and of the hydroxymethylation levels that we observe suggest they may play a role in the fine-tuning of targeted demethylation. Indeed, a model that seeks to explain DNA methylation dynamics at enhancers during differentiation has been proposed (Hon et al., 2014). This model suggests that at TF binding-rich enhancers, binding of TFs excludes DNMT1 activity, leading to their demethylation, whereas in TF binding-poor enhancers, TET2 protein is crucial in fine-tuning enhancer methylation in an oxidation-dependent manner. We suggest that a similar mechanism could be responsible for the targeted remodeling of these regions during reprogramming.

In contrast, AID does not have a role in targeted demethylation of pluripotency regulatory regions, but its absence results in widespread hypermethylated epialleles in iPSCs. This explains why AID-deficient iPSCs can be obtained, but cells with residual and persistent epigenetic memory may well behave aberrantly and unpredictably in potential future therapeutic settings, given their altered differentiation potential. It will be interesting to investigate further the differences in developmental potential between female and male iPSCs, which could impact on their use in basic and translational research. Hence, in a process where cells have to switch off a somatic expression program and upregulate a pluripotency network, global DNA demethylation seems to be important for the removal of epigenetic memory, while targeted demethylation at regulatory regions, and, in particular, at ESC super-enhancers, is crucial for the establishment of the pluripotent identity. Understanding and manipulating the two demethylation processes may result in improvements in the safety and the efficiency of obtaining robust, high quality iPSCs, prerequisites for therapeutic applications in regenerative medicine.

EXPERIMENTAL PROCEDURES

Reprogramming of MEFs to iPSCs

For each transfection, 0.8×10^6 MEFs were nucleofected using Amaxa Nucleofection Technology (Lonza AG; program A-023), according to the manufacturer's instructions, with 1 μg of each plasmid. Plasmids for reprogramming pB-TRE-OCKS, pBASE, and pB-CAG-rtTA were obtained from the Wellcome Trust Sanger Institute's plasmid repository. Reprogramming was performed in ESC medium (DMEM, 15% fetal bovine serum, 1% anti-anti, 1% MEM non-essential amino acids, 50 μM b-mercaptoethanol, and 10^3 U leukemia inhibitory factor [LIF]) in the presence or absence of doxycycline, in a 5% O_2 incubator. The medium was refreshed every other day. Colonies were picked on day 6 of reprogramming and expanded for at least 54 days. Cells were collected at different time points during reprogramming: mouse embryonic fibroblasts (MEFs), d6 fluorescence-activated cell sorted (FACS) refractory cells positive for Thy1 and negative for SSEA1 surface markers (d6T$^+$/S$^-$), and early reprogramming intermediates negative for Thy1 and positive for SSEA1 (d6T$^-$/S$^+$) as well as the reprogramming of individual colonies at intermediate-late stages of reprogramming (d21 iPSC and d29 iPSC) and established iPSCs (d60 iPSC).

All animal work carried out in this study is covered by a project license under the Animal (Scientific Procedures) Act 1986, and further regulated by the Babraham Institute Animal Welfare, Experimentation, and Ethics Committee.

ACCESSION NUMBERS

The accession number for the BS-seq and RNA-seq data reported in this paper is GEO: GSE69823.

SUPPLEMENTAL INFORMATION

Supplemental Information includes Supplemental Experimental Procedures and four figures and can be found with this article online at http://dx.doi.org/10.1016/j.celrep.2017.01.008.

AUTHOR CONTRIBUTIONS

I.M. conceived the project, designed and performed experiments, analyzed data, and wrote the manuscript; T.M.S. performed all PBAT libraries and data processing and provided helpful discussions; M.R.K. and J.S. performed WB experiments and karyotyping and provided technical assistance and helpful discussions; F.S. performed all immunofluorescence (IF) experiments; M.B. and S.B. performed all mass spectrometry experiments; F.K. and S.R.A. provided bioinformatics support; A.S.-P. performed all statistical analysis; W.D. performed all embryo work and provided helpful discussions for project design, and W.R. interpreted the data, provided helpful discussions for project design, and helped write the manuscript. All authors have interpreted the data and provided helpful discussions and read and approved the manuscript.

ACKNOWLEDGMENTS

We would like to thank Laura Biggins for help with data analysis and the Silva and Liu labs for sharing reprogramming protocols. We would like to thank José Silva and Cristina Rada for critical reading of the manuscript and helpful discussions. We would like to thank the members of W.R.'s and Rugg-Gunn's

(K) Density plots of methylation differences at ESC enhancer and SE from AidKO MEFs to iPSCs, shown by opaque plots. Overlaid by MMRP transparent gray density plot. Δ denotes a minimum 5% difference between data and MMRP profile p values shown are the result of a pairwise t test with a Benjamini-Hochberg correction.
(L) Proposed model to explain role of AID in DNA methylation dynamics during iPSC reprogramming.
See also Figure S4.

labs for helpful discussions and Dr. Veronique Juvin (veronique.juvin@sciartwork.com; http://www.sciartwork.com) for the design and creation of the model figure/graphical abstract. This work was funded by the Wellcome Trust (095645/Z/11/Z), BBSRC (BB/K010867/1), EU NoE Epigenesys, and FEBS (Long-term fellowship to I.M.). S.B. is a founder and shareholder in Cambridge Epigenetix, Ltd., an advisor and shareholder in Illumina, Inc., and a senior investigator of the Wellcome Trust (grant no. 099232/z/12/z). W.R. is a consultant and shareholder in Cambridge Epigenetix, Ltd.

Received: January 21, 2016
Revised: December 23, 2016
Accepted: January 6, 2017
Published: January 31, 2017

REFERENCES

Bhutani, N., Brady, J.J., Damian, M., Sacco, A., Corbel, S.Y., and Blau, H.M. (2010). Reprogramming towards pluripotency requires AID-dependent DNA demethylation. Nature *463*, 1042–1047.

Bhutani, N., Decker, M.N., Brady, J.J., Bussat, R.T., Burns, D.M., Corbel, S.Y., and Blau, H.M. (2013). A critical role for AID in the initiation of reprogramming to induced pluripotent stem cells. FASEB J. *27*, 1107–1113.

Bostick, M., Kim, J.K., Estève, P.O., Clark, A., Pradhan, S., and Jacobsen, S.E. (2007). UHRF1 plays a role in maintaining DNA methylation in mammalian cells. Science *317*, 1760–1764.

Brambrink, T., Foreman, R., Welstead, G.G., Lengner, C.J., Wernig, M., Suh, H., and Jaenisch, R. (2008). Sequential expression of pluripotency markers during direct reprogramming of mouse somatic cells. Cell Stem Cell *2*, 151–159.

Buganim, Y., Faddah, D.A., Cheng, A.W., Itskovich, E., Markoulaki, S., Ganz, K., Klemm, S.L., van Oudenaarden, A., and Jaenisch, R. (2012). Single-cell expression analyses during cellular reprogramming reveal an early stochastic and a late hierarchic phase. Cell *150*, 1209–1222.

Cacchiarelli, D., Trapnell, C., Ziller, M.J., Soumillon, M., Cesana, M., Karnik, R., Donaghey, J., Smith, Z.D., Ratanasirintrawoot, S., Zhang, X., et al. (2015). Integrative analyses of human reprogramming reveal dynamic nature of induced pluripotency. Cell *162*, 412–424.

Chen, H., Ma, H., Inuzuka, H., Diao, J., Lan, F., Shi, Y.G., Wei, W., and Shi, Y. (2013a). DNA damage regulates UHRF1 stability via the SCF(β-TrCP) E3 ligase. Mol. Cell. Biol. *33*, 1139–1148.

Chen, J., Liu, H., Liu, J., Qi, J., Wei, B., Yang, J., Liang, H., Chen, Y., Chen, J., Wu, Y., et al. (2013b). H3K9 methylation is a barrier during somatic cell reprogramming into iPSCs. Nat. Genet. *45*, 34–42.

Costa, Y., Ding, J., Theunissen, T.W., Faiola, F., Hore, T.A., Shliaha, P.V., Fidalgo, M., Saunders, A., Lawrence, M., Dietmann, S., et al. (2013). NANOG-dependent function of TET1 and TET2 in establishment of pluripotency. Nature *495*, 370–374.

Ding, J., Huang, X., Shao, N., Zhou, H., Lee, D.-F., Faiola, F., Fidalgo, M., Guallar, D., Saunders, A., Shliaha, P.V., et al. (2015). Tex10 coordinates epigenetic control of super-enhancer activity in pluripotency and reprogramming. Cell Stem Cell *16*, 653–668.

Doege, C.A., Inoue, K., Yamashita, T., Rhee, D.B., Travis, S., Fujita, R., Guarnieri, P., Bhagat, G., Vanti, W.B., Shih, A., et al. (2012). Early-stage epigenetic modification during somatic cell reprogramming by Parp1 and Tet2. Nature *488*, 652–655.

Ficz, G., Hore, T.A., Santos, F., Lee, H.J., Dean, W., Arand, J., Krueger, F., Oxley, D., Paul, Y.-L., Walter, J., et al. (2013). FGF signaling inhibition in ESCs drives rapid genome-wide demethylation to the epigenetic ground state of pluripotency. Cell Stem Cell *13*, 351–359.

Golipour, A., David, L., Liu, Y., Jayakumaran, G., Hirsch, C.L., Trcka, D., and Wrana, J.L. (2012). A late transition in somatic cell reprogramming requires regulators distinct from the pluripotency network. Cell Stem Cell *11*, 769–782.

Habib, O., Habib, G., Do, J.-T., Moon, S.-H., and Chung, H.M. (2014). Activation-induced deaminase-coupled DNA demethylation is not crucial for the generation of induced pluripotent stem cells. Stem Cells Dev. *23*, 209–218.

Habibi, E., Brinkman, A.B., Arand, J., Kroeze, L.I., Kerstens, H.H.D., Matarese, F., Lepikhov, K., Gut, M., Brun-Heath, I., Hubner, N.C., et al. (2013). Whole-genome bisulfite sequencing of two distinct interconvertible DNA methylomes of mouse embryonic stem cells. Cell Stem Cell *13*, 360–369.

Hon, G.C., Song, C.-X., Du, T., Jin, F., Selvaraj, S., Lee, A.Y., Yen, C.-A., Ye, Z., Mao, S.-Q., Wang, B.-A., et al. (2014). 5mC oxidation by Tet2 modulates enhancer activity and timing of transcriptome reprogramming during differentiation. Mol. Cell *56*, 286–297.

Hu, X., Zhang, L., Mao, S.-Q., Li, Z., Chen, J., Zhang, R.R., Wu, H.P., Gao, J., Guo, F., Liu, W., et al. (2014). Tet and TDG mediate DNA demethylation essential for mesenchymal-to-epithelial transition in somatic cell reprogramming. Cell Stem Cell *14*, 512–522.

Kim, D.H., Marinov, G.K., Pepke, S., Singer, Z.S., He, P., Williams, B., Schroth, G.P., Elowitz, M.B., and Wold, B.J. (2015). Single-cell transcriptome analysis reveals dynamic changes in lncRNA expression during reprogramming. Cell Stem Cell *16*, 88–101.

Kumar, R., DiMenna, L., Schrode, N., Liu, T.-C., Franck, P., Muñoz-Descalzo, S., Hadjantonakis, A.-K., Zarrin, A.A., Chaudhuri, J., Elemento, O., and Evans, T. (2013). AID stabilizes stem-cell phenotype by removing epigenetic memory of pluripotency genes. Nature *500*, 89–92.

Lee, D.-S., Shin, J.-Y., Tonge, P.D., Puri, M.C., Lee, S., Park, H., Lee, W.-C., Hussein, S.M.I., Bleazard, T., Yun, J.-Y., et al. (2014a). An epigenomic roadmap to induced pluripotency reveals DNA methylation as a reprogramming modulator. Nat. Commun. *5*, 5619.

Lee, H.J., Hore, T.A., and Reik, W. (2014b). Reprogramming the methylome: Erasing memory and creating diversity. Cell Stem Cell *14*, 710–719.

Leitch, H.G., McEwen, K.R., Turp, A., Encheva, V., Carroll, T., Grabole, N., Mansfield, W., Nashun, B., Knezovich, J.G., Smith, A., et al. (2013). Naive pluripotency is associated with global DNA hypomethylation. Nature Structural &#38. Mol. Biol. *20*, 311–316.

Lister, R., Pelizzola, M., Dowen, R.H., Hawkins, R.D., Hon, G., Tonti-Filippini, J., Nery, J.R., Lee, L., Ye, Z., Ngo, Q.-M., et al. (2009). Human DNA methylomes at base resolution show widespread epigenomic differences. Nature *462*, 315–322.

Mikkelsen, T.S., Hanna, J., Zhang, X., Ku, M., Wernig, M., Schorderet, P., Bernstein, B.E., Jaenisch, R., Lander, E.S., and Meissner, A. (2008). Dissecting direct reprogramming through integrative genomic analysis. Nature *454*, 49–55.

Nashun, B., Hill, P.W., and Hajkova, P. (2015). Reprogramming of cell fate: Epigenetic memory and the erasure of memories past. EMBO J. *34*, 1296–1308.

O'Malley, J., Skylaki, S., Iwabuchi, K.A., Chantzoura, E., Ruetz, T., Johnsson, A., Tomlinson, S.R., Linnarsson, S., and Kaji, K. (2013). High-resolution analysis with novel cell-surface markers identifies routes to iPS cells. Nature *499*, 88–91.

Papp, B., and Plath, K. (2013). Epigenetics of reprogramming to induced pluripotency. Cell *152*, 1324–1343.

Pastor, W.A., Aravind, L., and Rao, A. (2013). TETonic shift: Biological roles of TET proteins in DNA demethylation and transcription. Nat. Rev. Mol. Cell Biol. *14*, 341–356.

Polo, J.M., Anderssen, E., Walsh, R.M., Schwarz, B.A., Nefzger, C.M., Lim, S.M., Borkent, M., Apostolou, E., Alaei, S., Cloutier, J., et al. (2012). A molecular roadmap of reprogramming somatic cells into iPS cells. Cell *151*, 1617–1632.

Popp, C., Dean, W., Feng, S., Cokus, S.J., Andrews, S., Pellegrini, M., Jacobsen, S.E., and Reik, W. (2010). Genome-wide erasure of DNA methylation in mouse primordial germ cells is affected by AID deficiency. Nature *463*, 1101–1105.

Santos, F., Peat, J., Burgess, H., Rada, C., Reik, W., and Dean, W. (2013). Active demethylation in mouse zygotes involves cytosine deamination and base excision repair. Epigenetics Chromatin *6*, 39.

Seisenberger, S., Andrews, S., Krueger, F., Arand, J., Walter, J., Santos, F., Popp, C., Thienpont, B., Dean, W., and Reik, W. (2012). The dynamics of genome-wide DNA methylation reprogramming in mouse primordial germ cells. Mol. Cell *48*, 849–862.

Sharif, J., Muto, M., Takebayashi, S., Suetake, I., Iwamatsu, A., Endo, T.A., Shinga, J., Mizutani-Koseki, Y., Toyoda, T., Okamura, K., et al. (2007). The SRA protein Np95 mediates epigenetic inheritance by recruiting Dnmt1 to methylated DNA. Nature *450*, 908–912.

Shimamoto, R., Amano, N., Ichisaka, T., Watanabe, A., Yamanaka, S., and Okita, K. (2014). Generation and characterization of induced pluripotent stem cells from Aid-deficient mice. PLoS ONE *9*, e94735.

Shirane, K., Kurimoto, K., Yabuta, Y., Yamaji, M., Satoh, J., Ito, S., Watanabe, A., Hayashi, K., Saitou, M., and Sasaki, H. (2016). Global landscape and regulatory principles of DNA methylation reprogramming for germ cell specification by mouse pluripotent stem cells. Dev. Cell *39*, 87–103.

Soufi, A., Donahue, G., and Zaret, K.S. (2012). Facilitators and impediments of the pluripotency reprogramming factors' initial engagement with the genome. Cell *151*, 994–1004.

Sridharan, R., Tchieu, J., Mason, M.J., Yachechko, R., Kuoy, E., Horvath, S., Zhou, Q., and Plath, K. (2009). Role of the murine reprogramming factors in the induction of pluripotency. Cell *136*, 364–377.

Stadler, M.B., Murr, R., Burger, L., Ivanek, R., Lienert, F., Schöler, A., van Nimwegen, E., Wirbelauer, C., Oakeley, E.J., Gaidatzis, D., et al. (2011). DNA-binding factors shape the mouse methylome at distal regulatory regions. Nature *480*, 490–495.

Stadtfeld, M., Maherali, N., Breault, D.T., and Hochedlinger, K. (2008). Defining molecular cornerstones during fibroblast to iPS cell reprogramming in mouse. Cell Stem Cell *2*, 230–240.

Sugawa, F., Araúzo-Bravo, M.J., Yoon, J., Kim, K.-P., Aramaki, S., Wu, G., Stehling, M., Psathaki, O.E., Hübner, K., and Schöler, H.R. (2015). Human primordial germ cell commitment in vitro associates with a unique PRDM14 expression profile. EMBO J. *34*, 1009–1024.

Sun, J., Keim, C.D., Wang, J., Kazadi, D., Oliver, P.M., Rabadan, R., and Basu, U. (2013). E3-ubiquitin ligase Nedd4 determines the fate of AID-associated RNA polymerase II in B cells. Genes Dev. *27*, 1821–1833.

Surani, M.A. (2012). Cellular reprogramming in pursuit of immortality. Cell Stem Cell *11*, 748–750.

Tauber, M., and Fischle, W. (2015). Conserved linker regions and their regulation determine multiple chromatin-binding modes of UHRF1. Nucleus *6*, 123–132.

Tchieu, J., Kuoy, E., Chin, M.H., Trinh, H., Patterson, M., Sherman, S.P., Aimiuwu, O., Lindgren, A., Hakimian, S., Zack, J.A., et al. (2010). Female human iPSCs retain an inactive X chromosome. Cell Stem Cell *7*, 329–342.

von Meyenn, F., and Reik, W. (2015). Forget the parents: Epigenetic reprogramming in human germ cells. Cell *161*, 1248–1251.

von Meyenn, F., Iurlaro, M., Habibi, E., Liu, N.Q., Salehzadeh-Yazdi, A., Santos, F., Petrini, E., Milagre, I., Yu, M., Xie, Z., Kroeze, L.I., et al. (2016). Impairment of DNA methylation maintenance is the main cause of global demethylation in naive embryonic stem cells. Mol. Cell *62*, 848–861.

Wernig, M., Meissner, A., Foreman, R., Brambrink, T., Ku, M., Hochedlinger, K., Bernstein, B.E., and Jaenisch, R. (2007). In vitro reprogramming of fibroblasts into a pluripotent ES-cell-like state. Nature *448*, 318–324.

Wiench, M., John, S., Baek, S., Johnson, T.A., Sung, M.-H., Escobar, T., Simmons, C.A., Pearce, K.H., Biddie, S.C., Sabo, P.J., et al. (2011). DNA methylation status predicts cell type-specific enhancer activity. EMBO J. *30*, 3028–3039.

Yang, C.-S., Chang, K.Y., and Rana, T.M. (2014). Genome-wide functional analysis reveals factors needed at the transition steps of induced reprogramming. Cell Rep. *8*, 327–337.

Yoshida, Y., Takahashi, K., Okita, K., Ichisaka, T., and Yamanaka, S. (2009). Hypoxia enhances the generation of induced pluripotent stem cells. Cell Stem Cell *5*, 237–241.

Zvetkova, I., Apedaile, A., Ramsahoye, B., Mermoud, J.E., Crompton, L.A., John, R., Feil, R., and Brockdorff, N. (2005). Global hypomethylation of the genome in XX embryonic stem cells. Nat. Genet. *37*, 1274–1279.

Cell Reports, Volume *18*

Supplemental Information

Gender Differences in Global but Not Targeted Demethylation in iPSC Reprogramming

Inês Milagre, Thomas M. Stubbs, Michelle R. King, Julia Spindel, Fátima Santos, Felix Krueger, Martin Bachman, Anne Segonds-Pichon, Shankar Balasubramanian, Simon R. Andrews, Wendy Dean, and Wolf Reik

Figure S1

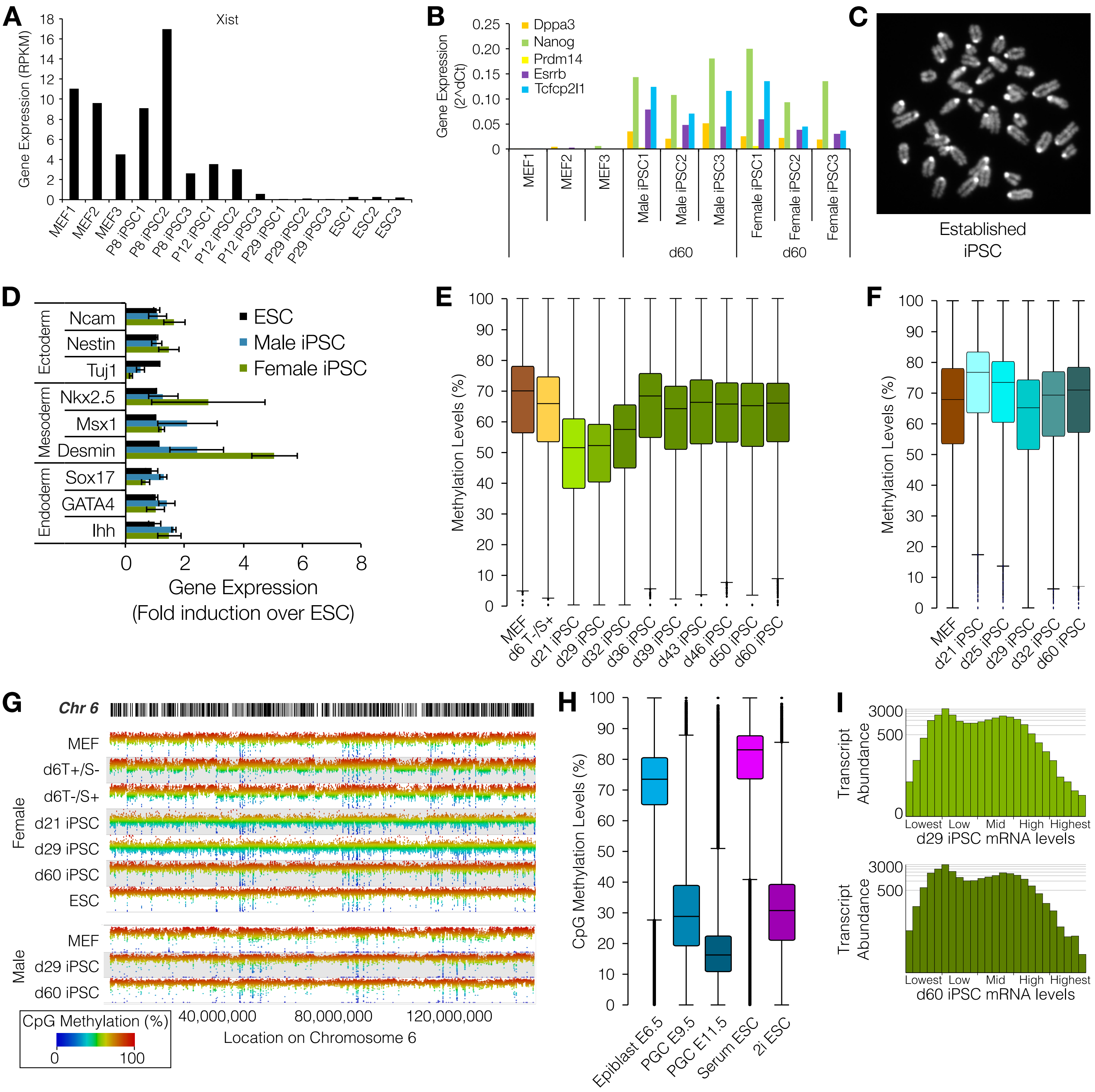
A
Xist
Gene Expression (RPKM)
MEF1 MEF2 MEF3 P8 iPSC1 P8 iPSC2 P8 iPSC3 P12 iPSC1 P12 iPSC2 P12 iPSC3 P29 iPSC1 P29 iPSC2 P29 iPSC3 ESC1 ESC2 ESC3
B
Dppa3
Nanog
Prdm14
Esrrb
Tcfcp2l1
Gene Expression (2^dCt)
MEF1 MEF2 MEF3 Male iPSC1 Male iPSC2 Male iPSC3 Female iPSC1 Female iPSC2 Female iPSC3
d60
d60
C
Established iPSC
D
Ectoderm
Ncam
Nestin
Tuj1
Mesoderm
Nkx2.5
Msx1
Desmin
Endoderm
Sox17
GATA4
Ihh
ESC
Male iPSC
Female iPSC
Gene Expression
(Fold induction over ESC)
E
Methylation Levels (%)
MEF d6 T-/S+ d21 iPSC d29 iPSC d32 iPSC d36 iPSC d39 iPSC d43 iPSC d46 iPSC d50 iPSC d60 iPSC
F
Methylation Levels (%)
MEF d21 iPSC d25 iPSC d29 iPSC d32 iPSC d60 iPSC
G
Chr 6
Female
MEF
d6T+/S-
d6T-/S+
d21 iPSC
d29 iPSC
d60 iPSC
ESC
Male
MEF
d29 iPSC
d60 iPSC
CpG Methylation (%)
0 100
40,000,000 80,000,000 120,000,000
Location on Chromosome 6
H
CpG Methylation Levels (%)
Epiblast E6.5 PGC E9.5 PGC E11.5 Serum ESC 2i ESC
I
Transcript Abundance
Lowest Low Mid High Highest
d29 iPSC mRNA levels
Transcript Abundance
Lowest Low Mid High Highest
d60 iPSC mRNA levels

Figure S1: iPSC reprogramming system and global DNA methylation (Related to Figure 1) (A) Expression profile (in RPKM) of *Xist* in each female iPSC clone and comparison with ESCs. (B) Expression profile (qPCR) of pluripotency genes upregulated in each iPSC clone and comparison with MEFs. (C) Representative karyotype of an established iPSC clone. (D) Differentiation markers expressed in embryoid bodies (EB) from each iPSC clone and comparison with EB from ESCs. (E-F) CpG methylation levels, as assessed by PBAT BS-Seq, at additional time-points of reprogramming of (E) female and (F) male cells. Plot displays the median (bar), inter-quartile range (box), and maximum and minimum (whiskers). (G) Methylation distribution and levels of each 50 CpG probe across chromosome 6, for all time-points during female and male cell reprogramming. (H) CpG methylation levels, as assessed by BS-Seq, for PGC development (Epiblast, PGCs E9.5 and E11.5) and ESCs (in serum and 2i conditions). Plot displays the median (bar), inter-quartile range (box), and maximum and minimum (whiskers). (I) Histogram of transcript abundance for genes showing different expression levels at d29 and d60 iPSCs.

Figure S2

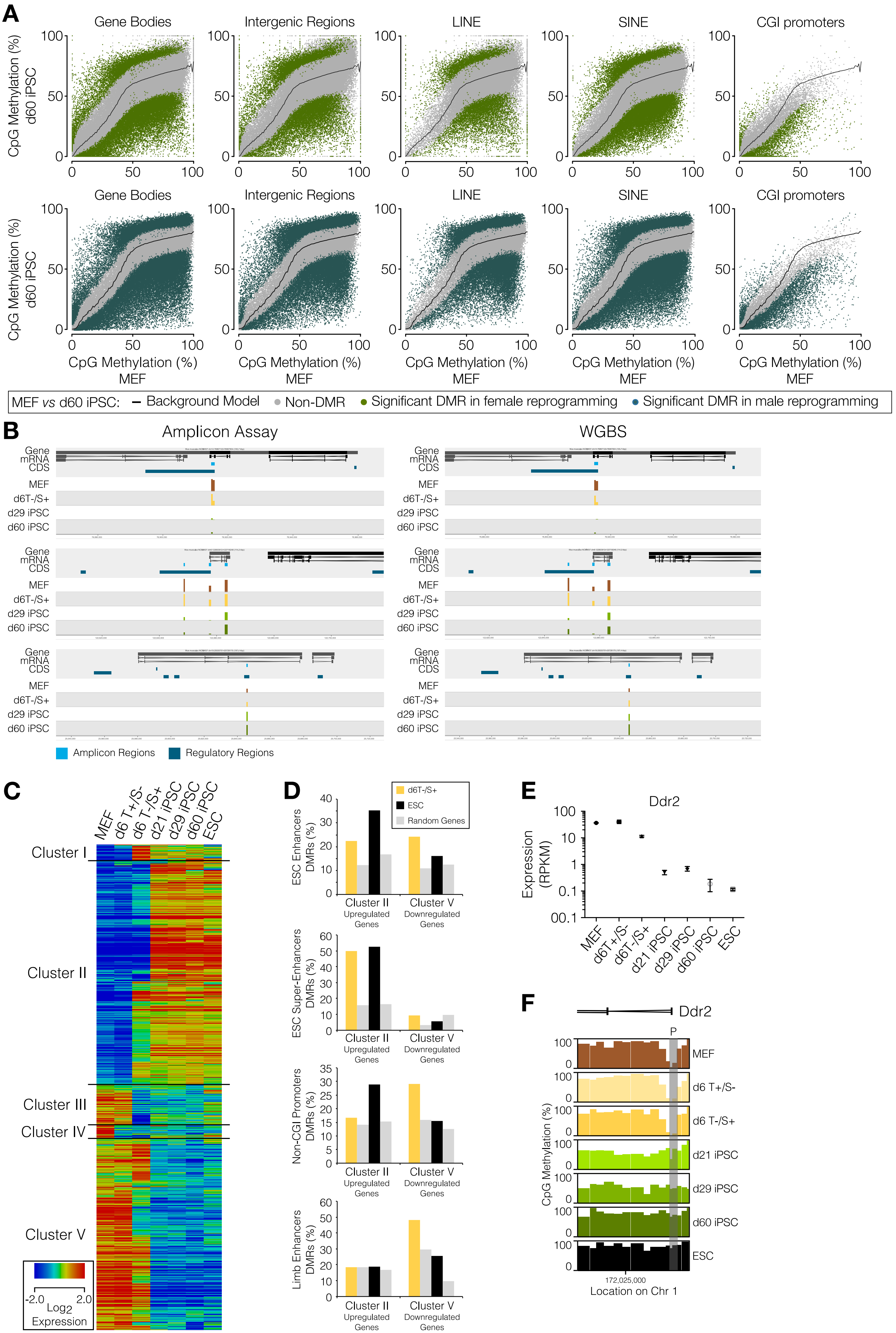

A
Gene Bodies
Intergenic Regions
LINE
SINE
CGI promoters
CpG Methylation (%) d60 iPSC
CpG Methylation (%) MEF
MEF vs d60 iPSC: Background Model
Non-DMR
Significant DMR in female reprogramming
Significant DMR in male reprogramming
B
Amplicon Assay
WGBS
Gene
mRNA
CDS
MEF
d6T-/S+
d29 iPSC
d60 iPSC
Amplicon Regions
Regulatory Regions
C
MEF
d6 T+/S-
d6 T-/S+
d21 iPSC
d29 iPSC
d60 iPSC
ESC
Cluster I
Cluster II
Cluster III
Cluster IV
Cluster V
-2.0
2.0
Log2 Expression
D
d6T-/S+
ESC
Random Genes
ESC Enhancers DMRs (%)
ESC Super-Enhancers DMRs (%)
Non-CGI Promoters DMRs (%)
Limb Enhancers DMRs (%)
Cluster II Upregulated Genes
Cluster V Downregulated Genes
E
Ddr2
Expression (RPKM)
100
10
1
0.1
00.1
MEF
d6T+/S-
d6T-/S+
d21 iPSC
d29 iPSC
d60 iPSC
ESC
F
Ddr2
P
MEF
d6 T+/S-
d6 T-/S+
d21 iPSC
d29 iPSC
d60 iPSC
ESC
CpG Methylation (%)
172,025,000
Location on Chr 1

Figure S2: Relationship between DNA methylation and gene expression (Related to Figure 2)

(A) Scatter plot of DNA methylation levels of individual probes genome-wide, showing different genomic features of MEFs and d60 iPSCs. Dots represent individual 50 CpG probes – significant differentially methylated regions (DMRs) are represented in green (female cell reprogramming) or blue (male cell reprogramming). Background model depicted as a black line. (B) Methylation profile for all regions confirmed by Amplicon-BS-Seq assay and comparison to WGBS profile. (C) Differentially expressed gene expression heat map. Clusters I to V represent different expression patterns during reprogramming. (D) Percentage of DMRs at regulatory regions, overlapping specific gene clusters. Shown are DMRs containing ESC enhancers and super-enhancers, non-CGI promoters and limb enhancers and their overlap with genes that are up-regulated during reprogramming (Cluster II) and genes that are down-regulated during reprogramming (Cluster V), comparing to random sets with the same number of genes, for d6T-/S+ and ESCs. (E) Expression profile (RPKM) of *Ddr2* gene. (F) Example of BS-seq profile for MEF gene *Ddr2* promoter region. Methylation levels of individual probes, between 0 and 100% are shown. Shadowed areas highlight promoter – P.

Figure S3

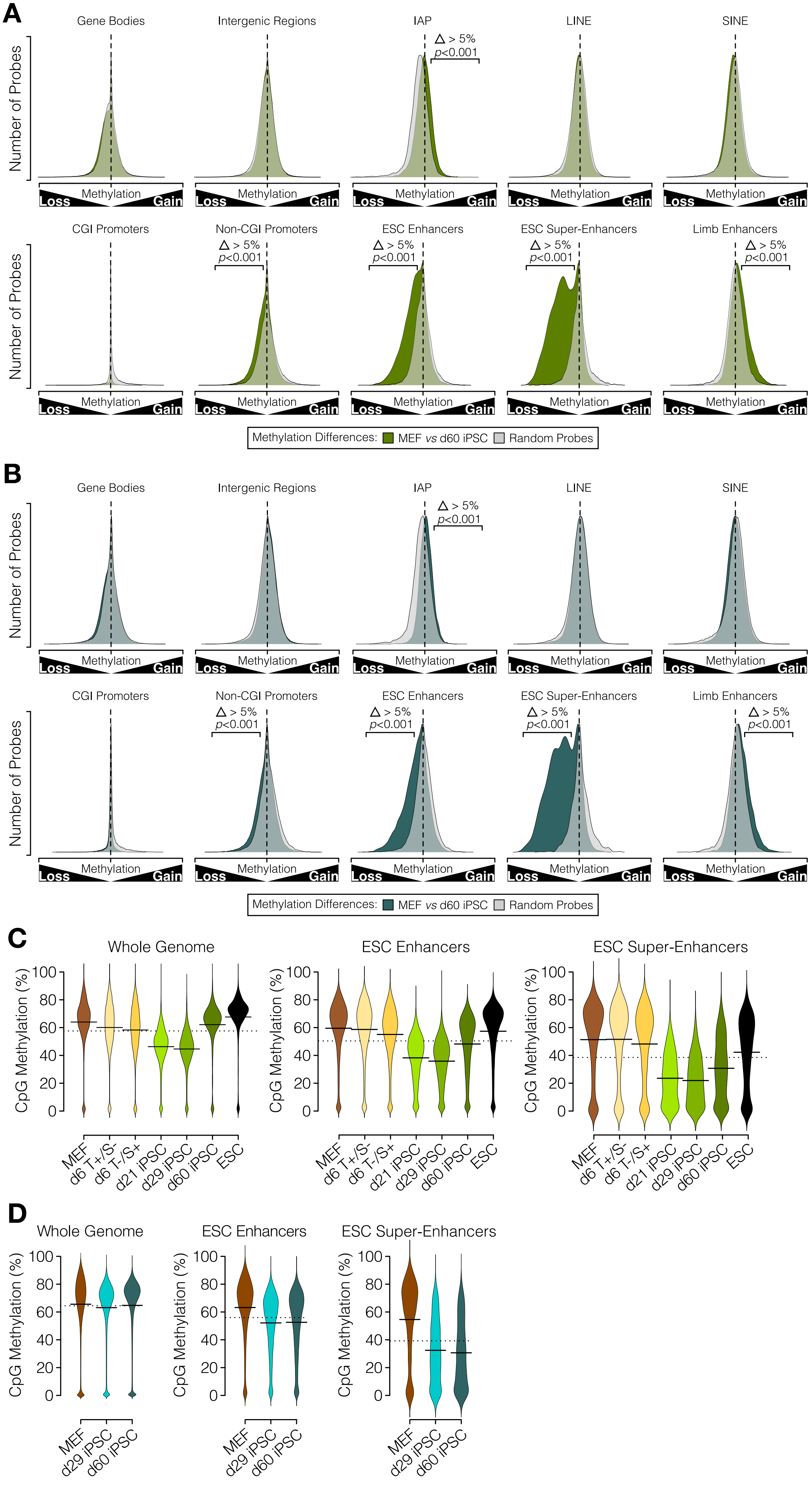
A
Gene Bodies
Intergenic Regions
IAP
LINE
SINE
CGI Promoters
Non-CGI Promoters
ESC Enhancers
ESC Super-Enhancers
Limb Enhancers
Number of Probes
Δ > 5%
p<0.001
Methylation
Loss
Gain
Methylation Differences: MEF vs d60 iPSC Random Probes
B
Gene Bodies
Intergenic Regions
IAP
LINE
SINE
CGI Promoters
Non-CGI Promoters
ESC Enhancers
ESC Super-Enhancers
Limb Enhancers
Number of Probes
Δ > 5%
p<0.001
Methylation
Loss
Gain
Methylation Differences: MEF vs d60 iPSC Random Probes
C
Whole Genome
ESC Enhancers
ESC Super-Enhancers
CpG Methylation (%)
0
20
40
60
80
100
MEF
d6 T+/S-
d6 T-/S+
d21 iPSC
d29 iPSC
d60 iPSC
ESC
D
Whole Genome
ESC Enhancers
ESC Super-Enhancers
CpG Methylation (%)
MEF
d29 iPSC
d60 iPSC

Figure S3: DNA demethylation dynamics at different genomic features (Related to Figure 3)

(A-B) Density plots of methylation differences for different genomic features from MEFs to d60 iPSCs, shown by opaque dark green (A-female) or dark blue (B-male) plots. Overlaid by MMRP transparent grey density plot. Δ denotes a minimum 5% difference between data and MMRP profile. *p*-values shown are the result of a pairwise *t*-test with a Benjamini-Hochberg correction. (C-D) CpG methylation levels for whole genome, ESC enhancers and super-enhancers, for female (C) and male (D) reprogramming. Beanplots show the density of loci for each methylation value.

Figure S4

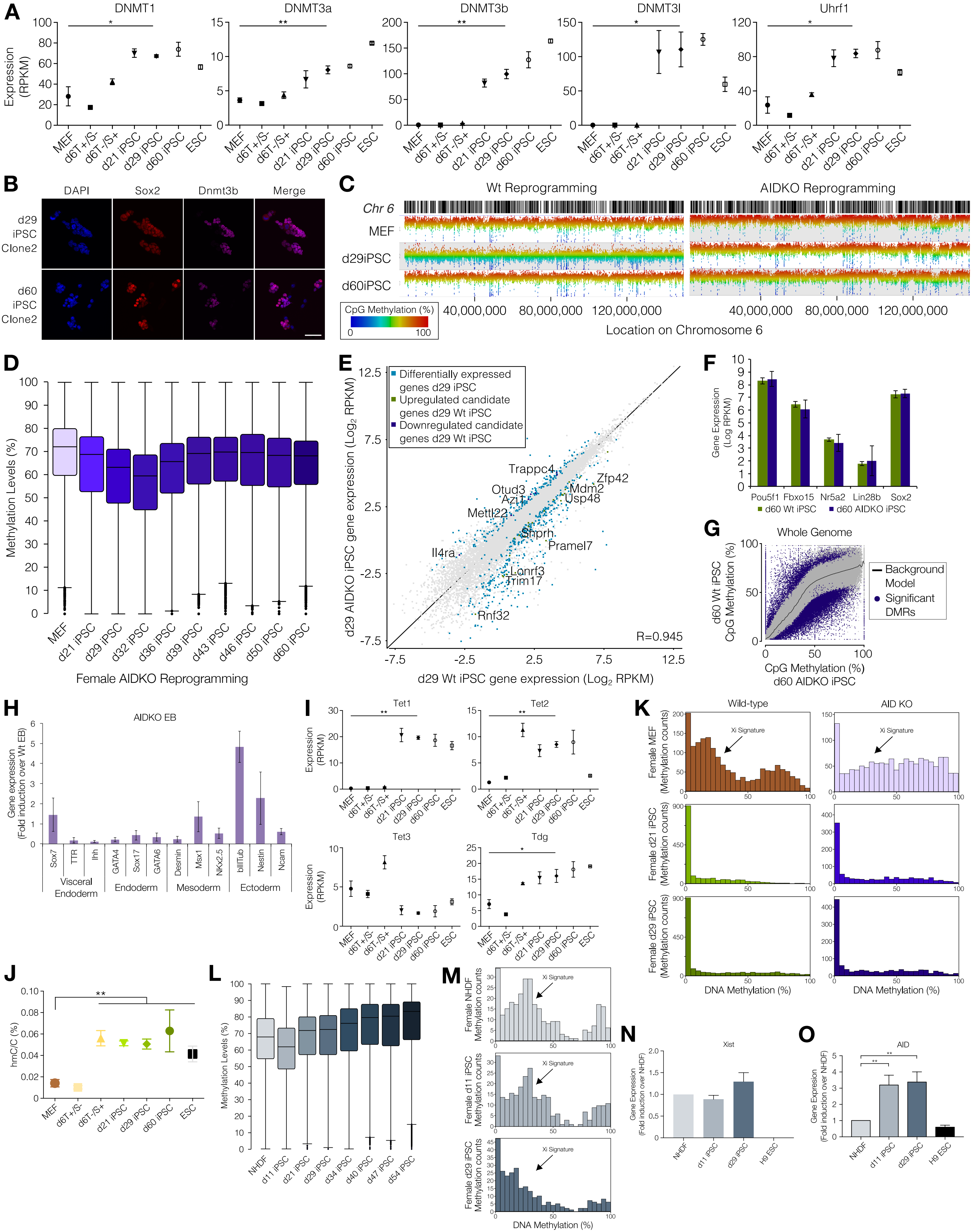
A
DNMT1
DNMT3a
DNMT3b
DNMT3l
Uhrf1
Expression (RPKM)
MEF
d6T+/S-
d6T-/S+
d21 iPSC
d29 iPSC
d60 iPSC
ESC
B
DAPI
Sox2
Dnmt3b
Merge
d29 iPSC Clone2
d60 iPSC Clone2
C
Wt Reprogramming
AIDKO Reprogramming
Chr 6
MEF
d29iPSC
d60iPSC
CpG Methylation (%)
40,000,000
80,000,000
120,000,000
Location on Chromosome 6
D
Methylation Levels (%)
MEF
d21 iPSC
d29 iPSC
d32 iPSC
d36 iPSC
d39 iPSC
d43 iPSC
d46 iPSC
d50 iPSC
d60 iPSC
Female AIDKO Reprogramming
E
Differentially expressed genes d29 iPSC
Upregulated candidate genes d29 Wt iPSC
Downregulated candidate genes d29 Wt iPSC
d29 AIDKO iPSC gene expression (Log2 RPKM)
d29 Wt iPSC gene expression (Log2 RPKM)
Trappc4
Zfp42
Otud3
Mdm2
Azi1
Usp48
Mettl22
Shprh
Pramel7
Il4ra
Lonrf3
Trim17
Rnf32
R=0.945
F
Gene Expression (Log RPKM)
Pou5f1
Fbxo15
Nr5a2
Lin28b
Sox2
d60 Wt iPSC
d60 AIDKO iPSC
G
Whole Genome
d60 Wt iPSC CpG Methylation (%)
CpG Methylation (%) d60 AIDKO iPSC
Background Model
Significant DMRs
H
AIDKO EB
Gene expression (Fold induction over Wt EB)
Sox7
TTR
Ihh
GATA4
Sox17
GATA6
Desmin
Msx1
NKx2.5
bIIITub
Nestin
Ncam
Visceral Endoderm
Endoderm
Mesoderm
Ectoderm
I
Tet1
Tet2
Tet3
Tdg
Expression (RPKM)
K
Wild-type
AID KO
Xi Signature
Female MEF (Methylation counts)
Female d21 iPSC (Methylation counts)
Female d29 iPSC (Methylation counts)
DNA Methylation (%)
J
hmC/C (%)
L
Methylation Levels (%)
NHDF
d11 iPSC
d21 iPSC
d29 iPSC
d34 iPSC
d40 iPSC
d47 iPSC
d54 iPSC
M
Female NHDF Methylation counts
Female d11 iPSC Methylation counts
Female d29 iPSC Methylation counts
Xi Signature
DNA Methylation (%)
N
Xist
Gene Expression (Fold induction over NHDF)
NHDF
d11 iPSC
d29 iPSC
H9 ESC
O
AID
Gene Expresion (Fold induction over NHDF)

Figure S4: Mechanisms and global DNA demethylation in different reprogramming systems (Related to Figure 4)

(A) Expression profile (in RPKM) of *Dnmt1, 3a, 3b, 3l* and *Uhrf1*. *p*-values shown are the result of two-tailed *t*-tests from MEFs to d29 iPSCs. Data are represented as mean ± SEM. (B) Immunofluorescence (IF) of staining of Wt iPSCs at d29 and d60. Shown are representative IF for DAPI, SOX2 and DNMT3b for one iPSC clone. (C) Methylation distribution and levels of each 50 CpG probe across chromosome 6, for MEFs, d29 and d60 iPSCs during AIDKO MEF reprogramming. (D) CpG methylation levels, as assessed by PBAT BS-Seq, at additional time-points of reprogramming of AIDKO cells. Plot displays the median (bar), inter-quartile range (box), and maximum and minimum (whiskers). (E) Scatter plot of RNA expression levels in d29 Wt and AIKO iPSCs. Differentially expressed genes (blue), upregulated (green) and downregulated (purple) candidate genes. (F) Expression profile (RPKM) of pluripotency genes in Wt and AIDKO d60 iPSCs. (G) Scatter plot of DNA methylation levels of individual probes genome-wide, showing whole genome of d60 Wt and AIDKO iPSCs. Individual dots represent individual 50 CpG probes, DMRs are represented in purple and probes that do not differ between samples in grey. Background model depicted as a black line. (H) Differentiation markers expressed in embryoid bodies (EB) from AIDKO iPSCs, shown are fold induction values compared to Wt iPSCs. (I) Expression profile (in RPKM) of *Tet1-3* and *Tdg*. *p*-values shown are the result of two-tailed *t*-tests from MEF to d29 iPSCs. Data are represented as mean ± SEM. (J) Global 5hmC levels, measured by LC-MS. Results are expressed as percentage of total cytosine. Data are represented as mean ± SEM. *p*-values shown are the result of an ANOVA multiple-comparison test with Dunnett's multiple comparison test: $p<0.0001$. (K) Distribution of methylation levels across CpG islands on the X chromosome in indicated cell types and time-points during reprogramming, based on BS-Seq data. The arrow indicates the Xi-specific DNA methylation signature. (L) CpG methylation levels, as assessed by PBAT BS-Seq, during reprogramming of human cells. Plot displays the median (bar), inter-quartile range (box), and maximum and minimum (whiskers). (M) Distribution of methylation levels across CpG islands on the X chromosome in indicated cell types during human iPSC reprogramming, based on PBAT BS-Seq data. The arrow indicates the Xi-specific DNA methylation signature. (N-O) Expression profile (qPCR) of (N) *Xist* and (O) *Aid* during human female reprogramming. Data are represented as mean ± SEM. *p*-values shown are the result of an ANOVA multiple-comparison test with Dunnett's multiple comparison test: $p<0.01$.

Supplemental Experimental Procedures

Cell culture

ESC and iPSC culture – TMBD10 ESCs (derived by the gene targeting facility at the Babraham Institute from C57Bl/6 mice) and iPSCs were grown in ESCs medium (DMEM, 15% fetal bovine serum, 1% Anti-Anti, 1% MEM Non-Essential Amino Acids, 50 µM 2-mercaptoethanol and 10^3 U LIF). ES and iPS cells were grown either in complete ESC medium in gelatinized dishes or differentiated for 11 days into embryoid bodies via LIF removal and suspension culture.

Isolation of MEF - 13.5 to 14.5 days post coitum (d.p.c.) Wild-type Oct4-GFP or AidKO Oct4-GFP embryos were decapitated, eviscerated, dissociated with 0.25% trypsin, 0.1% EDTA and plated onto MEF medium (DMEM, 10% FBS, 1% Anti-Anti, 1% MEM Non-Essential Amino Acids and 50 µM 2-mercaptoethanol). The Oct4/GFP strain carries a transgene comprising 18kb of the endogenous murine Oct4 locus including downstream regulatory elements and the endogenous Oct4 promoter from which green fluorescent protein (GFP) is expressed (Yeom et al., 1996; Yoshimizu et al., 1999), the AidKO Oct4-GFP has been previously described and characterized (Popp et al., 2010). All animal work carried out in this study is covered by a project license under the Animal (Scientific Procedures) Act 1986, and further regulated by the Babraham Institute Animal Welfare, Experimentation, and Ethics Committee.

Rescue experiments – For each transfection, $0.8x10^6$ MEFs were nucleofected with reprogramming plasmids plus an empty vector, Wt or Catalytic-mutant AID. Cells were selected with blasticidin for 5 days. Picked and expanded colonies were collected at d21 and d29 and DNA extracted to be used in LC-MS analysis and protein extracts used in WB.

Reprogramming of human Fibroblasts to iPSCs

Reprogramming was performed by infecting $3.0x10^5$ normal human dermal fibroblasts (NHDF - GIBCO) with CytoTune®-iPS 2.0 Sendai Reprogramming Kit (Invitrogen), according to manufacturer's instruction, at an MOI of 1. Cells were maintained in fibroblast medium (DMEM, 10% FBS, 1% Anti-Anti, 1% MEM Non-Essential Amino Acids and 50 µM 2-mercaptoethanol) for five days. Infected cells were then replated onto feeder dishes and maintained in KSR medium (Advanced DMEM:F12, 20% KSR, 1% Anti-Anti, 1% MEM Non-Essential Amino Acids, 25 µM 2-mercaptoethanol and 4ng/ml bFGF). Medium was replenished everyday. Cells were collected at different time-points during reprogramming by FACS (NHDF, d11, d21, d29) or manually (d34, d40, d47 and d54).

Fluorescence-activated cell sorting (FACS)

For cell sorting, mouse cells were harvested and incubated with antibodies against Thy1.2 (PE, eBioscience) and SSEA-1 (eFluor 660, eBioscience), human cells were incubated with antibodies against CD13 (PE, BD Pharmigen) and SSEA-4 (Alexa Fluor 647, BD Pharmigen) for 30 min. Cells were washed in a 2% FBS/PBS solution and passed through a 50µm cell strainer to achieve single-cell suspension. Cells were stained with DAPI just prior to sorting. Appropriate negative and positive controls were used to assess optimal FACS conditions. Cell sorting was performed using an Influx cell sorter instrument (BD Biosciences) and cells were collected for downstream applications.

Karyotyping

Cells were grown in gelatinised dishes overnight and colchicine (0.04 μg/ml) was added to the medium for 1 hour. Subsequently, cells were trypsinised to single cells, pelleted and resuspended in hypotonic buffer KCl 0.075M. After 5-minute incubation at 37ºC, cells were fixed in ice-cold acetic/methanol 1:3 at 4ºC for 2 hours. Cells were dropped onto chilled slides, stained with DAPI for 2 minutes and observed under an Olympus BX61 fluorescence microscope. At least 80 chromosome spreads were counted and each cell line was classified as having normal or abnormal karyotypes.

Liquid chromatography – Mass-spectrometry
Genomic DNA (a minimum of 100ng) was incubated with DNA Degradase Plus (Zymo Research) at 37°C for 4 h. Detection of global mC and hmC levels was carried out on an AB Sciex Triple Quad 6500 mass spectrometer as previously described (Bachman et al., 2014). Results are expressed as percentage of total cytosine.

DNA and RNA extraction and BS-Seq Library Preparation
For each time point 3 biological replicates from 3 independent experiments were analysed. These consisted of MEFs, a day 6 FACS sorted refractory population (d6 Thy1+/SSEA1-: d6T+/S-) and cells prone to reprogramming (d6 Thy1-/SSEA1+: d6T-/S+), Dox-independent iPSCs at intermediate-late stages of reprogramming (d21 and d29 iPSCs) and established iPSCs (d60 iPSC), and a C57BL/6 background ESC line (TMBD10 - ESCs). Genomic DNA and total RNA was prepared using AllPrep DNA/ RNA mini kit (QIAGEN). For BS-Seq library preparation, DNA samples were fragmented by sonication (Covaris) and adaptor ligated using Illumina supplied methylated adaptors and NEBnext library preparation kit. Subsequently, DNA was bisulphite-treated using the TrueMethyl kit (Cambridge Epigenetix), according to the manufacturer's instructions. Final library amplification (11 cycles) was done using Kapa Uracil Plus (Kapa Biosytems), after which the libraries were bead-purified using 1x XP Ampure beads (Beckman Coulter). Libraries were sequenced on Illumina HiSeq.
For PBAT libraries, DNA samples were bisulphite converted using the EZ-DNA methylation direct kit. First strand synthesis was conducted as previously described (Smallwood et al., 2014), however, using F oligo 6N (CTACACGACGCTCTTCCGATCTNNNNNN) instead of a biotinylated 8N oligo. Samples were exonuclease treated. Second strand synthesis was conducted using a 8R oligo 6N (TGCTGAACCGCTCTTCCGATCTNNNNNN). Samples were amplified for 10 cycles using Kapa HotStart (Kapa Biosystems).

RNASeq Library Preparation
For each sample mRNA was purified from 50 ng of total RNA, using a Dynabeads® mRNA Purification Kit (Life Technologies) according to the manufacturer's instructions. This RNA was subsequently used for library preparation as described previously (Lin Wang et al., 2011). Libraries were amplified (14 cycles) and purified using 0.8x XP Ampure beads. Libraries were sequenced on Illumina HiSeq 2500.

BS-Seq Analysis
Raw sequence reads were trimmed to remove both poor quality calls and adapters using Trim Galore (Babraham Institute Bioinformatics). For PBAT libraries the 5' end of reads were also trimmed. Sequences were mapped to the mouse NCBIM37 genome using Bismark (Krueger and Andrews, 2011), and CpG methylation calls were extracted and analysed with SeqMonk and R. For PBAT analysis, 35k probes were designed across the whole genome and global methylation levels obtained. For BS-Seq, in order to obtain 3-fold coverage, replicates from

each time-point were pooled together. 50 CpG dinucleotide-containing probes were design across the whole genome and methylation over a given genomic feature was calculated by averaging the individual methylation levels of CpGs across these probes, only probes with reads in at least 10 CpGs were taken forward for analysis. CpG island (CGI) annotations were used based on pull down experiments (Illingworth et al., 2010). Promoters were defined as the region -1 kb to +500 bp of the transcription start site as annotated in NCBIM37, and were subdivided into CGI and non-CGI promoters based on whether they overlapped with a CGI. Repeat annotations were extracted from the UCSC RepeatMasker track (NCBIM37). ESC enhancer and super-enhancer (Whyte et al., 2013) and limb enhancer (Shen et al., 2012) coordinates were described previously. Background model was obtained by calculating the global mean for the ending methylation value for each starting methylation value. Afterwards, a binomial test was used on individual probes for their ending methylation measures against the mean for their starting methylation, to obtain p-value<0.05 DMRs. Differences in methylation at specific features were calculated and compared to a set of methylation matched random probes (MMRP). Probes from the reference sample whole genome with ±5% methylation were picked to create a subset of MMRP with an equal number of probes to the feature analysed. p-values in each feature were calculated using a pairwise t-test with a Benjamini-Hochberg correction. Targeted demethylation was considered only in features that deviated from the MMRP profile by at least 5% and had a significant p-value after Benjamini-Hochberg correction. Publically available datasets for oocyte, 2-cell and 4-cell embryos, ICM, Epiblast (Lu Wang et al., 2014), Epiblast E6.5, PGC E9.5, PGC E11.5 (Seisenberger et al., 2012), serum grown ESC, 2i grown ESCs (Ficz et al., 2013) were included in this analysis where indicated.

BS-Seq Amplicon assay

For BS-seq library preparation DNA samples were bisulphite-treated using the Epitect Kit (QIAGEN), according to the manufacturer's instructions. DNA was PCR-amplified using specific primers for 35 cycles. All PCR products for each sample were pooled together and bead-purified using 1x XP Ampure beads (Beckman Coulter). This pooled library was then amplified for additional 8 cycles using iPCRTag (Quail et al., 2012) primers. Libraries were sequenced on Illumina MiSeq. Primer design was carried out using MethPrimer (Li and Dahiya, 2002), and a 33bp adapter for high-throughput sequencing was added to the primer. Selected primers amplified specific pluripotency gene regulatory regions, highly significant DMRs at ESC enhancers or super-enhancers and limb enhancers.

RNASeq Analysis

RNA-Seq data was mapped to the mouse genome assembly (NCBIM37) using TopHat and gene models from Ensembl release 61. The number of reads mapping to each annotated mRNA transcript was quantified and counts were used in DESeq and Seqmonk to assess differentially expressed genes, during reprogramming. Expression clusters were defined by performing hierarchical clustering of transcripts based on a Pearson's correlation across all samples, and selecting groups which had a correlation coefficient greater than 0.7. Counts corrected for total read count with length correction (reads per kilobase per million, RPKM) were used to plot graphs and perform statistical tests using Prism 6 (GraphPad Software Inc., San Diego, CA, USA).

Gene expression analysis by qPCR

Total RNA was prepared using the Qiagen AllPrep DNA/RNA mini kit according to the manufacturers' instructions. cDNA was obtained by reverse transcription of 0.5 µg of RNA using SuperScript III reverse-transcriptase kit with random hexamer primers (Invitrogen), diluted 1:50 and used as a template in quantitative real-time PCR (qPCR) with a Bio-Rad C1000 Thermal Cycler. The relative abundance of each gene of interest was normalized to the average Ct of two housekeeping genes with consistent Ct values over all samples (Atp5b and Hsp90ab1) and calculated using the ddCt method.

Western blot (WB) analysis

For WB analysis, whole cell extracts were resolved by SDS-PAGE and blotted on PVDF membranes. Membranes were blocked in TBS-Tween (5% milk) and incubated overnight at 4°C with the indicated antibodies. After incubation with secondary antibodies, HRP conjugates were detected using an enhanced chemiluminescence kit (ECL Plus, Amersham Biosciences). The following primary antibodies were used for Western: Dnmt1 (ab92453, Abcam), UHRF1 (Santa Cruz Biotechnology, sc-98817). Secondary antibodies used: HRP-conjugated anti-mouse, anti-rabbit (Santa Cruz Biotechnology, sc-2005, sc-2004) and anti-rat (GE healthcare, NA935).

Immunofluorescence, microscopy and image analysis

Cells were fixed in 2% paraformaldehyde for 30 min. After fixation cells were permeabilised with PBS+0.5% Triton X-100 for 1 hour and blocked overnight. Cells were incubated with anti-Dnmt3b (ab13604, Abcam), Sox2 (ab97959, Abcam) for 1 hour, washed and incubated with secondary antibodies (Alexa Fluor). Nuclei were stained with DAPI. Single optical slices were captured with a Zeiss LSM 780 or phase contrast Nikon TS100.

Supplemental References

Bachman, M., Uribe-Lewis, S., Yang, X., Williams, M., Murrell, A., and Balasubramanian, S. (2014). 5-Hydroxymethylcytosine is a predominantly stable DNA modification. Nat Chem *6*, 1049–1055.

Illingworth, R.S., Gruenewald-Schneider, U., Webb, S., Kerr, A.R.W., James, K.D., Turner, D.J., Smith, C., Harrison, D.J., Andrews, R., and Bird, A.P. (2010). Orphan CpG Islands Identify Numerous Conserved Promoters in the Mammalian Genome. PLoS Genet *6*, e1001134.

Krueger, F., and Andrews, S.R. (2011). Bismark: a flexible aligner and methylation caller for Bisulfite-Seq applications. Bioinformatics *27*, 1571–1572.

Li, L.-C., and Dahiya, R. (2002). MethPrimer: designing primers for methylation PCRs. Bioinformatics *18*, 1427–1431.

Quail, M.A., Otto, T.D., Gu, Y., Harris, S.R., Skelly, T.F., McQuillan, J.A., Swerdlow, H.P., and Oyola, S.O. (2012). Optimal enzymes for amplifying sequencing libraries. Nat. Methods *9*, 10–11.

Smallwood, S.A., Lee, H.J., Angermueller, C., Krueger, F., Saadeh, H., Peat, J., Andrews, S.R., Stegle, O., Reik, W., Kelsey, G., 2014. Single-cell genome-wide bisulfite sequencing for assessing epigenetic heterogeneity. Nat. Methods 11, 817–820. doi:10.1038/nmeth.3035

Shen, Y., Yue, F., McCleary, D.F., Ye, Z., Edsall, L., Kuan, S., Wagner, U., Dixon, J., Lee, L., Lobanenkov, V.V., Ren, B., 2012. A map of the cis-regulatory sequences in the mouse genome. Nature 488, 116–120. doi:10.1038/nature11243

Whyte, W.A., Orlando, D.A., Hnisz, D., Abraham, B.J., Lin, C.Y., Kagey, M.H., Rahl, P.B., Lee, T.I., Young, R.A., 2013. Master Transcription Factors and Mediator Establish Super-Enhancers at Key Cell Identity Genes. Cell 153, 307–319.

doi:10.1016/j.cell.2013.03.035

Wang, Lin, Si, Y., Dedow, L.K., Shao, Y., Liu, P., Brutnell, T.P., 2011. A Low-Cost Library Construction Protocol and Data Analysis Pipeline for Illumina-Based Strand-Specific Multiplex RNA-Seq. PLoS ONE 6, e26426. doi:10.1371/journal.pone.0026426.s015

Wang, Lu, Zhang, J., Duan, J., Gao, X., Zhu, W., Lu, X., Yang, L., Zhang, J., Li, G., Ci, W., Li, W., Zhou, Q., Aluru, N., Tang, F., He, C., Huang, X., Liu, J., 2014. Programming and Inheritanceof Parental DNA Methylomes in Mammals. Cell 157, 979–991. doi:10.1016/j.cell.2014.04.017

Yeom, Y.I., Fuhrmann, G., Ovitt, C.E., Brehm, A., Ohbo, K., Gross, M., Hubner, K., Schöler, H.R., 1996. Germline regulatory element of Oct-4 specific for the totipotent cycle of embryonal cells. Development 122, 881–894.

Yoshimizu, T., Sugiyama, N., De Felice, M., Yeom, Y.I., Ohbo, K., Masuko, K., Obinata, M., Abe, K., Schöler, H.R., Matsui, Y., 1999. Germline-specific expression of the Oct-4/green fluorescent protein (GFP) transgene in mice. Dev. Growth Differ. 41, 675–684.
